# Characterization and GC–MS-Based Identification of Possible PVC Degradation Pathways of Bacteria for Sustainable Microplastic Bioremediation

**DOI:** 10.3390/microorganisms14092114

**Published:** 2026-09-21

**Authors:** Nadia Hussain, Alishba Saif, Fatima Muccee, Muhammad Imran Afzal, Wardah Shahid, Amal H. I. Al Haddad

**Affiliations:** 1 Department of Pharmaceutical Sciences, Al Ain University, Al Ain Campus, Abu Dhabi 64141, United Arab Emirates; nadia.hussain@aau.ac.ae; 2 Department of Pharmaceutical Sciences, College of Pharmacy, Al Ain University, Al Ain Campus, Al Ain 64141, United Arab Emirates; 3 School of Biochemistry and Biotechnology, University of Punjab, Lahore 52254, Pakistan; alishbasaif57@gmail.com (A.S.); ranaaimran007@gmail.com (M.I.A.); 4 Department of Biochemistry, Faculty of Biological Sciences, Quaid-e-Azam University, Islamabad 45320, Pakistan; wardah@qau.edu.pk or wardahshahid@ymail.com; 5 Chief Operations Office, Sheikh Shakhbout Medical City (SSMC), PureHealth, Abu Dhabi 11001, United Arab Emirates; ahhaddad@ssmc.ae

**Keywords:** polyvinyl chloride, *Bacillus flexus*, molecular identification, bioremediation, weight loss assay

## Abstract

Polyvinyl chloride (PVC), due to its persistent nature and the hazardous effects associated with chlorine content, poses global challenges like accelerated environmental pollution, public health crises and an increased economic burden. Its sustainable decontamination from the environment could be possible with the assistance of bacteria. The current study was initiated to achieve this sustainable goal. PVC-degrading bacteria were isolated via the serial dilution method using minimal salt medium (MSM) supplemented with PVC as a sole carbon source and characterized via 16S RNA gene sequencing analysis, growth curve and PVC strip weight loss assays, antibiotic sensitivity profiling, FTIR and GC–MS-based metabolic profiling. Five isolates, *Achromobacter xylosoxidans* SBBPVC1, *Bacillus licheniformis* SBBPVC2, *Pseudomonas citronellolis* SBBPVC3, *P. stutzeri* SBBPVC4 and *Bacillus flexus* SBBPVC5 were identified. All were fast-growing, with PVC degradation efficiency ranging between 20–50% per 30 days. The FTIR spectral comparison with the control clearly indicated peak variations in regions 1100–1250, 1370, 1460 and 1720–1740 cm^−1^, corresponding to C-O and C-C, CH_3_, CH_2_ and C=O bond vibrations, validating PVC degradation in the presence of bacteria. GC–MS-based identification of four esters, i.e., 1-methyl heptyl, isooctyl, adipic acid, methyl octyl and octyl myristate esters, along with aliphatic alcohols and aldehydes, confirmed the degradation of PVC and its use as a carbon source by bacteria. Bacteria explored in current study could be used as bioremediating agents for PVC decontamination of the environment.

## 1. Introduction

For more than 70 years, plastics have shaped our daily lives and industries because they are resilient, cost-effective and versatile [1,2]. According to an estimate of the Environment Protection Agency (EPA), the annual plastic waste and national solid waste generation of Pakistan is 3.3 million tons and 49.6 million tons, respectively. Plastics constitute roughly 9 to 11% of total solid waste [3]. Each year, there is an average increase of 8 kg per capita in plastic waste generation. Higher localized totals are demonstrated in urban centers, including Lahore, which generates approximately 0.65 kg of total municipal solid waste per capita per day [4]. Approximately 70% of plastic waste remains unmanaged due to the unavailability of major infrastructure. The Indus River functions as one of the main global pathways transferring plastic waste into the marine ecosystem [5]. According to the EPA, approximately 55 billion single-use plastic bags are used annually. Plastic polymers have been widely used in daily life, agriculture and in the chemical industry during the past century. Their extensive use and poor management have resulted in plastic waste accumulation in water bodies and canals, blocking drainage systems and hindering wastewater (WW) treatment procedures [6].

After polyethylene (PE) and polypropylene (PP), polyvinyl chloride (PVC) constitutes the third most widely produced plastic in the world. It has intrinsic persistence due to phthalates, heavy metals, salts and high chlorine content [7]. Due to this toughness, it has potential applications in plumbing, flooring, toys, furniture, construction materials production and packaging [8]. The strong covalent bonds, high hydrophobicity and molecular weight make PVC decomposition difficult. These attributes result in the poor management of plastic waste, causing health hazards and mortality [9].

Conventional methods of degrading PVC include chemical, mechanical, and physical methods such as landfilling, recycling and burning. Landfilling is a low-cost strategy but requires larger areas and leads to slow decomposition due to anaerobic conditions [10]. UV irradiation can render plastics more prone to microbial attack, but it is energy-intensive, costly and unfavorable for massive applications [11]. In addition, these strategies generate toxic byproducts like vinyl monomers, CO_2_, chlorofluorocarbons (CFCs) and dioxins. Recycling, which converts plastics into reusable materials, is a superior alternative, but this approach exploits massive amounts of energy [12]. Thermal treatment of PVC requires significant amounts of energy, which renders it costly [4]. Overall, each approach has drawbacks, highlighting the urgent need for scalable and sustainable plastic waste management solutions [10].

Bacterial and fungal-based plastic degradation has emerged as a climate-friendly and sustainable mode of plastic remediation versus traditional physicochemical and mechanical approaches [11]. Landfilling, UV irradiation and the incineration breakdown of plastic can result in harmful gas emissions in the ecosystem, high costs, a decrease in soil fertility and the risk of partial degradation [12]. As a consequence, biologically regulated plastic degradation is clearly a safer and more sustainable choice due to cost effectiveness, reduced land disturbance and complete plastic degradation [13].

Microbes initiate the plastic degradation process by releasing hydrolyzing enzymes. Post hydrolysis of polymers, microbes tend to absorb degradation products and exploit them as a carbon source. Studies in the literature document lipase, laccase, amidase, oxidase, esterase, manganese peroxidase (MnP), alpha beta hydrolases (ABH), lignin peroxidase (LiP), carboxylesterases, proteases and cutinases as PVC-, polyethylene (PE)- and polyurethane (PU)-degrading enzymes [14,15,16,17]. Enzymes associated with fungi, like *Phanerochaete chrysosporium*, have demonstrated promise in high molecular-weight polymer degradation [18].

*Pseudomonas citronellolis*, *P. chlororaphis*, *Bacillus flexus*, *B. subtilis*, *B. albus*, *Chelatococcus daeguensis* and *Streptomyces gobitricini* have been documented as PVC-degrading bacteria [15,19,20]. These bacteria have been tested for PVC microplastics remediation via experimental work.

While these advancements show promise, slow growth rates and poor efficiency limit the effectiveness of previously documented bacteria. Additionally, the absence of hydrolysable ester linkages makes PVC uniquely resistant to biological breakdown. Prior research suggests that microbial consortia can degrade PVC compounds and their associated plasticizers. However, clear evidence of complete polymer breakdown remains elusive, as existing claims rely primarily on observable morphological and physicochemical changes. Furthermore, PVC degradation-associated enzymes and metabolic pathways remain unknown. Given the urgent need for sustainable management of plastic waste, the current project aims to isolate and characterize indigenous bacteria from plastic-contaminated soil in Pakistan and analyze their PVC biodegradation efficiency.

## 2. Materials and Methods

### 2.1. Sample Collection and Preparation of PVC Films

The soil sample was collected from a plastic waste site in Multan, Pakistan, for the isolation of plastic-degrading bacteria (Supplementary Materials, Figure S1A,B). PVC cling film (Supplementary Materials, Figure S1C) was purchased commercially, cut into 2 cm^2^ strips, and sterilized with 70% ethanol under aseptic conditions.

### 2.2. Isolation of Bacteria

The bacteria were isolated using the serial dilution method using minimal salt medium (MSM). The MSM was composed of 4.55 g KH_2_PO_4_, 11.94 g Na_2_HPO_4_, 1 g NH_4_Cl, 0.5 g MgSO_4_, 0.005 g CaCl_2_, 0.002 g FeSO_4_, 0.001 g MnSO_4_, 0.002 g ZnSO_4_ and 15 g agar-agar, dissolved in distilled water (1 L). All chemicals were purchased from Alkemist Supplier, Pakistan. Following debris removal by sifting, 1 g of soil was suspended in Ringer’s solution (25 mL) and incubated overnight (37 °C and 150 rpm). Afterwards, 200 µL of the original solution and subsequent dilutions (10^−1^ to 10^−6^) were placed onto MSM agar plates that contained sterile PVC strips. PVC strips were purchased from Al-Fatah, Shadman, Lahore, Pakistan. For 48–72 h, plates were incubated at 37 °C. Potential PVC-degrading bacteria were identified from colonies that showed growth. The following formula was used to determine the colony-forming units (CFUs):

$$CFU/mL=\frac{\left(Number\;of\;colonies)\times (Dilution\;factor\right)}{Volume\;of\;culture\;plated}$$

Individual colonies were sub-cultured in MSM broth and incubated overnight at 37 °C with shaking at 150 rpm. Cell pellets were obtained by centrifugation (6000× *g*, 4 °C and 10 min), using centrifuge machine (Thermo Scientific, Osterode am Harz, Germany, Cat No. 75007200) and 30% glycerol stocks were prepared and stored at −20 °C.

### 2.3. Morphological and Biochemical Characterization

Morphological characterization was conducted by streaking on MSM agar plates followed by overnight incubation, noting the size, shape, margin, color and texture of the colony. The Gram reaction of each colony was also examined using the Gram staining technique [21].

Biochemical profiling included catalase, mannitol fermentation, hydrogen cyanide (HCN) production, indole acetic acid (IAA) production and sugar fermentation tests for three carbohydrates, i.e., glucose, sucrose and lactose.

### 2.4. Molecular Identification

The genomic DNA of bacteria was extracted using the organic extraction method involving the use of phenol:chloroform:isoamyl alcohol (25:24:1) [22]. To identify bacterial strains and species, 16S rRNA gene amplification was performed using previously reported primers, i.e., forward 5′-AGAGTTTGATCCTGGCTCAG-3′ and reverse 5′-AAGGAGGTGATCCAGCCGCA-3′, with melting temperatures (Tm) of 55 and 60 °C, respectively [23]. Primers were purchased from Asketch, Lahore, Pakistan). The amplicon size was 1500 bp. PCR amplification was performed in a 25 μL reaction volume consisting of 12.5 μL master mix (Biopharmaceuticals, Centre of Excellence in Molecular Biology (CEMB), Lahore, Pakistan, CAT No. #PZM1010), 1.0 μL of forward primer, 1.0 μL of reverse primer, 1.0 μL of template DNA, 0.5 μL Taq polymerase (Thermo FischerScientific, CAT No. EPO404) and 9.0 μL nuclease-free water. Using a programmed thermocycler (Bio-Rad T100TM, Bio-Rad Laboratories, Inc., Hercules, CA, USA), thermal cycling was performed as follows: initial denaturation at 95 °C for 5 min; 35 cycles of denaturation at 95 °C for 40 s; annealing at 58 °C for 40 s; and extension at 72 °C for 1 min, followed by the final extension step. The amplified products were kept at 4 °C after PCR until they could be examined further. PCR products were visualized by agarose gel electrophoresis to confirm DNA amplification. Amplicons were analyzed via Sanger sequencing at Macrogen Inc., Seoul, Republic of Korea.

### 2.5. Bioinformatics Analysis

Utilizing the BLAST tool (https://blast.ncbi.nlm.nih.gov/, accessed on 5 January 2025), sequence identification was carried out by comparing each isolate to the NCBI GenBank nucleotide database to determine the percentage similarity and validate the taxonomic identity [24]. Sequences that were verified were added to the NCBI Genbank database (https://www.ncbi.nlm.nih.gov/nucleotide/, accessed on 5 January 2025) to have the accession numbers assigned [25]. CLUSTAL Omega (https://www.ebi.ac.uk/jdispatcher/msa/clustalo, accessed on 5 January 2025) was used to align the sequences for phylogenetic analysis and gaps were manually eliminated to produce ungapped alignments [26]. The Neighbor-Joining method in MEGA version 11 was used to build a phylogenetic tree; branch support was evaluated using bootstrap analysis (1000 repetitions) [27,28].

### 2.6. Growth Curve Analysis

In order to assess the bacterial growth in the presence of PVC, optical density (OD_600_) was measured over time. The overnight-grown culture (200 µL), with OD_600_ = 0.1, was added to five sterile 100 mL Erlenmeyer flasks that contained autoclaved MSM broth (30 mL) and PVC strips; one flask was used as an uninoculated control. Flasks were incubated at 37 °C with shaking at 150 rpm for 24 h. OD_600_ was recorded at multiple time points (0–72 h) to monitor growth. All experiments were performed in triplicate and growth curves were plotted to assess the bacterial proliferation rate.

### 2.7. Assessment of PVC Degradation Potential of Bacteria

#### 2.7.1. Weight Loss Assay

The 2 cm^2^ strip of PVC was cut and washed with distilled water. Strips were sterilized by immersion in ethanol (70%) for 10 min and air dried. To enhance biodegradability, strips were pretreated with 5 M NaOH at 60–80 °C for 2–3 h, then washed, re-sterilized and dried under aseptic airflow. A precision analytical balance (XP205, Mettler-Toledo International, Inc., Greifensee, Switzerland) was used to record the initial weights of the PVC strips. Sterile, pre-treated strips were shifted to autoclaved flasks (06) holding sterile MSM broth (10 mL).

The inoculum (200 µL of 30% bacterial glycerol stock) was added to each of the five flasks; one served as an un-inoculated control. All flasks were incubated (37 °C and 150 rpm) for 30 days [4], with weekly media replenishment under sterile conditions. Post incubation, strips were retrieved, washed with distilled water, sterilized with 70% ethanol (Sigma-Aldrich, St. Louis, MO, USA, CAS No. 64-17-5) and treated with 10% SDS (Sigma-Aldrich CAS No. 151-21-3, US) to remove biofilm. After thorough washing and drying, final weights were recorded. The experiment was performed in biological (02) and technical (02) replicates. PVC degradation was calculated using the formula:

$$\%\;Degradation\;of\;PVC=\frac{\left(initial\;weight-final\;weight\right)}{\left(Initial\;weight\right)}\;\times 100$$

To prevent additional microbial activity, both the treated and control strips were aseptically stored at 4 °C. Fourier transform infrared spectroscopy (FTIR) was used to evaluate structural alterations in the polymer matrix.

For the weight loss assay, a comprehensive two-part analysis was conducted, i.e., one-sample *t* test (baseline comparison) and ANOVA and post hoc ranking.

#### 2.7.2. FTIR Characterization of PVC Strip

Following bacterial treatment, FTIR spectroscopy was conducted to assess the chemical structure and possible changes in the PVC strips. It detected structural alterations in the polymer and identified functional groups. Bacteria-treated and control PVC strips from the weight loss assay were sent for FTIR analysis. Spectral analysis was performed over a wavelength range of 4000 to 400 cm^−1^ [29].

#### 2.7.3. PVC Topography via SEM Analysis

To predict the variations in the PVC strips’ topography treated with NaOH and bacteria versus the abiotic control, scanning electron microscopy (SEM) (Japan Electron Optics Laboratory, Tokyo, Japan, JSM-6480 LV) was employed. For this purpose, control, NaOH- and *P. citronellolis* SBBPVC3-treated strips, after the 30 days of incubation, were sent to the Central Research Lab, University of Lahore (UOL), Pakistan, for SEM analysis.

#### 2.7.4. GC–MS Analysis

Two bacterial isolates, SBBPVC1 and SBBPVC3, were selected for metabolic profiling based on high PVC degradation potential. Growth was achieved in MSM medium supplemented with PVC strips under shaking conditions at 37 °C and 150 rpm until the exponential phase. Extracellular and intracellular metabolites were extracted following a previously described protocol [30]. For extracellular metabolites, culture supernatants were collected and mixed with methanol in a 2:1 ratio. Mixtures were centrifuged (10,000× *g* for 20 min and 4 °C) and the upper methanol-rich layer (~3 mL) was transferred to sterile glass vials and incubated at 50 °C for one week to enable complete solvent evaporation.

For intracellular metabolites, the bacterial pellet was washed with 0.9% NaCl (Chemie-R, Dawn Scientific, Medford, NJ, USA, CAS No. 7647-14-5) and resuspended in a mixture of deionized water, methanol and chloroform (800 µL each) (BIORAYS, Lahore, Pakistan). The samples were sonicated for 1 h at 4 °C to ensure effective cell lysis, followed by centrifugation at 10,000× *g* for 20 min. Three distinct layers were formed, from which the upper aqueous (polar) and lower organic (non-polar) phases were carefully collected and dried. Both extracellular and intracellular metabolite extracts were sent to the Textile Testing International Laboratory, Quaid-e-Azam Industrial Estate.

For identification of the metabolites of bacterial PVC degradation, the NIST Library was consulted. Considering the possibility of the NIST library of not containing every possible compound, and for expert verification, we also performed comparisons in the literature [31,32]. For the comparisons, we considered the current study’s GC–MS spectra with the fragmentation patterns of metabolic intermediates of PVC that were documented in previous work.

### 2.8. Antibiotic Sensitivity Profiling

Stock solutions of the antibiotics gentamicin and chloramphenicol (1 mg/mL), ampicillin, erythromycin, and ciprofloxacin (0.1 mg/mL) were prepared to assess the antibiotic susceptibility of PVC-degrading bacterial isolates. Sterile filter paper discs (1 cm diameter) were loaded with 5, 10 and 15 µL of each antibiotic to create a concentration gradient. All antibiotics were purchased from PHARMASOL (Pvt) Ltd., Lahore, Pakistan. Petri plates containing solidified LB agar were divided into four quadrants labeled as control, 5, 10 and 15 µL. Each plate was inoculated with 100 µL of the freshly grown bacterial culture and spread evenly. Antibiotic discs were aseptically placed on the respective quadrants, while one disc without antibiotic served as the negative control. After sealing with parafilm, plates were incubated (37 °C for 24 h) in an inverted position and observed for zones of inhibition. The diameters (mm) of clear zones were measured around each disc to detect the sensitivity of isolates. Disc diameter was not included in the reported inhibition-zone measurements; zones were measured from the disc edge outward.

### 2.9. Statistical Analysis

All statistical analyses were performed using Python (version 3.x) with SciPy (version 1.x) statistical package. The one-factor-at-a-time (OFAT) statistical design was used in the current study analysis. The significance level (α) was set at 0.05 for all tests. A one-sample *t* test was used to compare each isolate’s degradation, growth and inhibition-zone values against their respective fixed baselines (0% degradation, 0 mm inhibition and media-blank OD_600_). These baselines are single, non-varying reference points rather than experimental distributions; thus, two-sample rank-based tests (Mann–Whitney U, Wilcoxon signed-rank) are not statistically appropriate here: with a control of zero variance, such tests degenerate and return non-informative *p*-values, regardless of the true effect size.

## 3. Results

### 3.1. Isolation of Bacteria

Bacterial colonies were observed on original and dilution (10^−1^ and 10^−2^) plates following the incubation of 48 h. The remaining plates did not demonstrate any growth. Bacterial CFUs were calculated (Supplementary Materials, Figure S2 and Table S1). *A. xylosoxidans* SBBPVC1, *Pseudomonas citronellolis* SBBPVC3 and *P. stutzeri* SBBPVC4 were isolated from the plate with dilution factor 10^−1^. *Bacillus licheniformis* SBBPVC2 and *B. flexus* SBBPVC5 were isolated from the plate with dilution factor 10^−2^.

### 3.2. Morphological Characterization

The morphology of the bacteria was studied in terms of size, color, edges and texture (Supplementary Materials, Table S2). *A. xylosoxidans* SBBPVC1, *B. licheniformis* SBBPVC2, *P. citronellolis* SBBPVC3 and *B. flexus* SBBPVC5 were circular, with pale yellow, off-white, yellowish and white to pale yellow-colored colonies, respectively. The *P. stutzeri* SBBPVC4 colony was creamy and irregular. *A. xylosoxidans* SBBPVC1 and *P. citronellolis* SBBPVC3 exhibited entire edges. *B. licheniformis* SBBPVC2, *P. stutzeri* SBBPVC4 and *B. flexus* SBBPVC5 showed undulate, irregular and wavy edges, respectively. *A. xylosoxidans* SBBPVC1, *P. citronellolis* SBBPVC3 and *P. stutzeri* SBBPVC4 demonstrated a Gram-negative character and *B. licheniformis* SBBPVC2 and *B. flexus* SBBPVC5 were Gram-positive.

### 3.3. Biochemical Characterization

A variety of enzymatic and metabolic activities were identified by the biochemical analysis of the five PVC-degrading bacteria. When hydrogen peroxide was added, bubbles quickly formed, indicating that all isolates had catalase activity. All isolates showed mannitol fermentation when they grew on MSA and changed color from red to yellow. The orange-red coloring of the picric acid filter paper indicated that *P. citronellolis* SBBPVC3 and *P. stutzeri* SBBPVC4 were producing HCN, while *A. xylosoxidans* SBBPVC1 showed moderate activity and the other isolates were negative. The development of a pink color following the addition of Kovac’s reagent indicated the strong production of IAA in *A. xylosoxidans* SBBPVC1, *B. licheniformis* SBBPVC2 and *P. citronellolis* SBBPVC3. *P. stutzeri* SBBPVC4 and *B. flexus* SBBPVC5 showed weak IAA production. *A. xylosoxidans*, *P. stutzeri* and *B. flexus* fermented glucose in sugar fermentation tests; however, neither *B. licheniformis* nor *P. citronellolis* did. All five isolates demonstrated positive lactose fermentation, as evidenced by a change in growth medium color. All bacteria except *B. flexus* showed sucrose fermentation (Supplementary Materials, Figures S3 and S4).

### 3.4. Molecular Characterization

Genomic DNA was successfully extracted from all bacterial isolates and PCR amplicons were visualized on agarose gel (Supplementary Materials, Figure S5). A total of five PVC-degrading bacterial strains were isolated and identified based on top BLAST sequence matches. These isolates showed closest similarity to *A. xylosoxidans*, *B. licheniformis* strain, *P. citronellolis*, *P. stutzeri* strain and *B. flexus*. The strains were designated as SBBPVC1, SBBPVC2, SBBPVC3, SBBPVC4, SBBPVC5, respectively; “SBB” denotes the name of the department (School of Biochemistry and Biotechnology) where the study was conducted. “PVC” represents polyvinyl chloride and the numerical suffix indicates the order of isolation. The accession numbers for the identified strains are assigned as: PV794637 (*A. xylosoxidans* SBBPVC1), PV797396 *(B*. *licheniformis* SBBPVC2), PV797336 (*P. citronellolis* SBBPVC3), PV797410 (*P. stutzeri* SBBPVC4) and PV797561 *(B. flexus* SBBPVC5).

### 3.5. Phylogenetic Analysis

**Figure 1:**
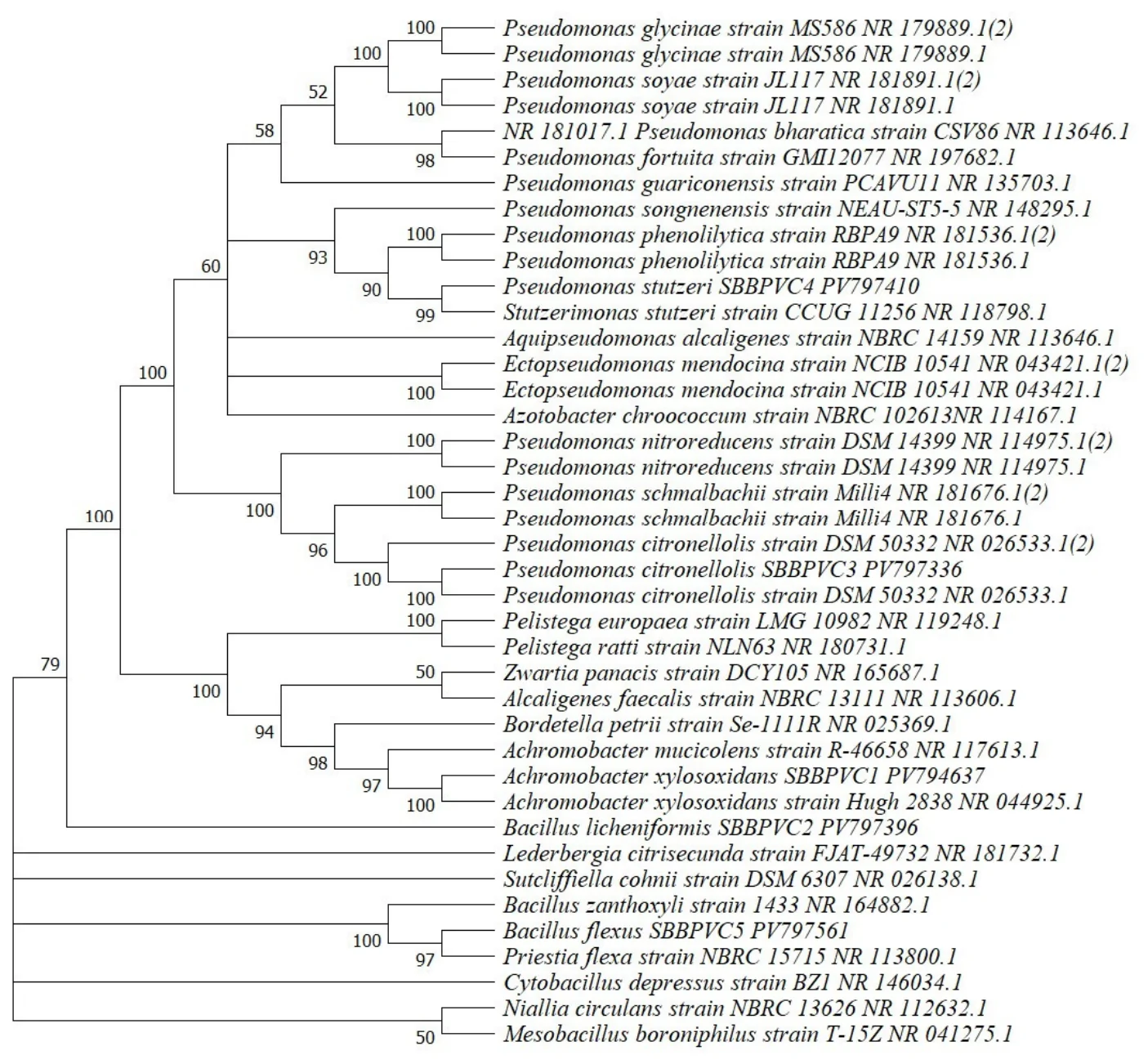
Phylogenetic tree construction for current study of PVC-degrading bacteria via MEGA 11 software.

*A. xylosoxidans* SBBPVC1 and *A. xylosoxidans* strain Huph 2838 originated from the same branch point, with a 100 bootstrap value reflecting their evolutionary proximity. *B. licheniformis* SBBPVC2 showed a similarity with the species of *Pseudomonas*, *Pelistega*, *Zwartia*, *Alcaligenes*, *Bordetella* and *Achromobacter* via a bootstrap value of 100. *P. citronellolis* SBBPVC3 shared a clade with *P. citronellolis* strains DSM via a bootstrap value of 100, indicating a close relationship. *P. stutzeri* SBBPVC4 showed evolutionary linkage with *Stutzerimonas stutzeri* strain CCUG 11256NR because both shared the same clade, with a bootstrap value of 99. *B. flexus* SBBPVC5 and *Priestia flexa* NBRC 15715 shared same the branch point, with a bootstrap value of 97. Both isolates shared a cluster with *B. zanthoxyli* strain 1433, via a bootstrap value of 100, confirming their relatedness (Figure 1).

### 3.6. Growth Curve Analysis

**Figure 2:**
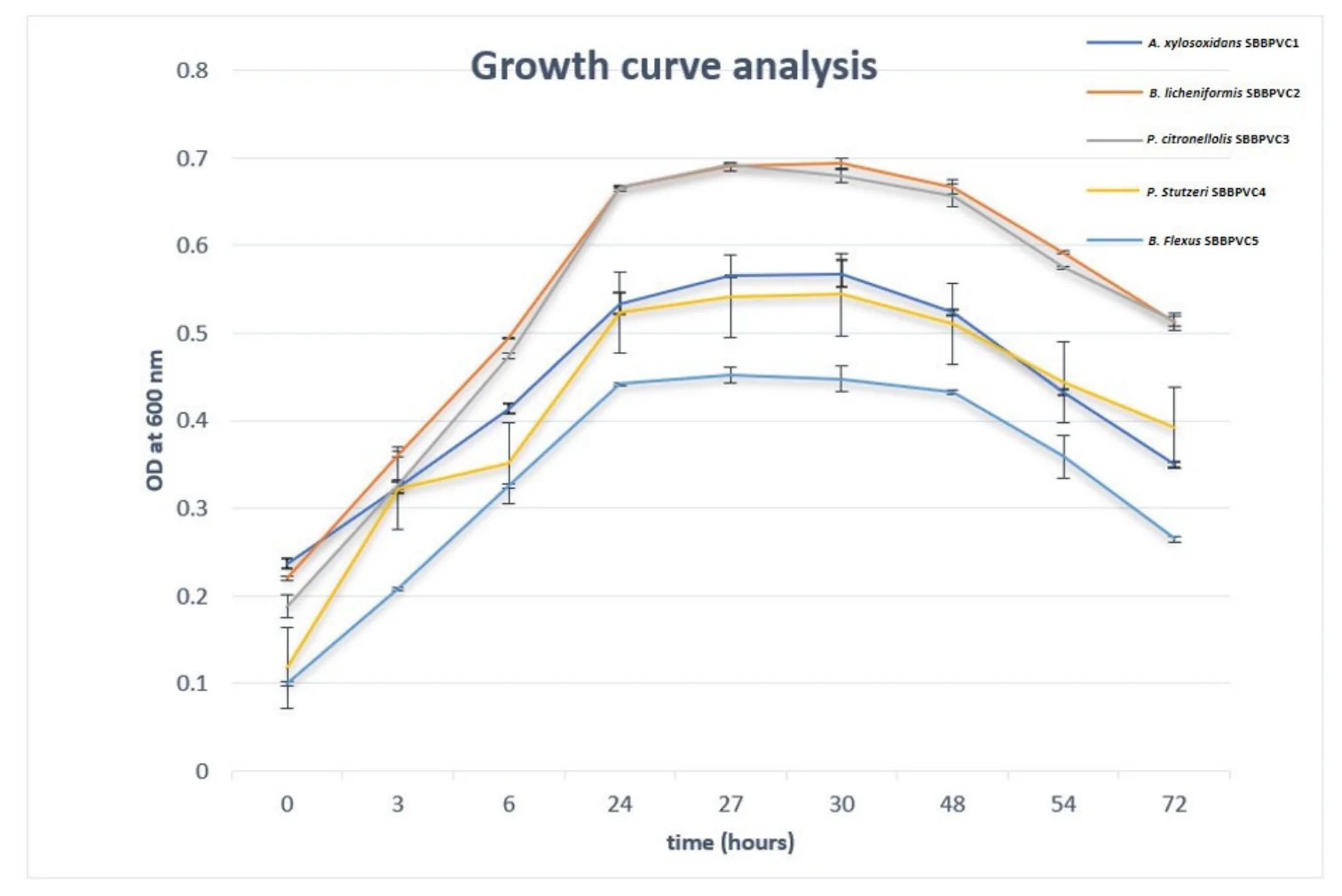
Growth curve analysis of PVC-degrading bacterial isolates.

All bacterial isolates were fast-growing, with the log phase initiating after 3 h in *A. xylosoxidans* SBBPVC1, *B. licheniformis* SBBPVC2 and *P. citronellolis* SBBPVC3, and after 6 h in *P. stutzeri* SBBPVC4 and *B. flexus* SBBPVC5. In all these bacteria, the log phase lasted 24 h. In all isolates, the stationary phase started after 24 h and lasted 30 h, except *P. citronellolis* SBBPVC3, where the stationary phase ended at 27 h. After that, the death phase began and OD_600_ values gradually decreased after 30 h onwards (Figure 2). During the stationary and death phases, *B. licheniformis* SBBPVC2 notably maintained comparatively steady OD_600_ values (Supplementary Materials, Tables S3 and S4).

**Figure 3:**
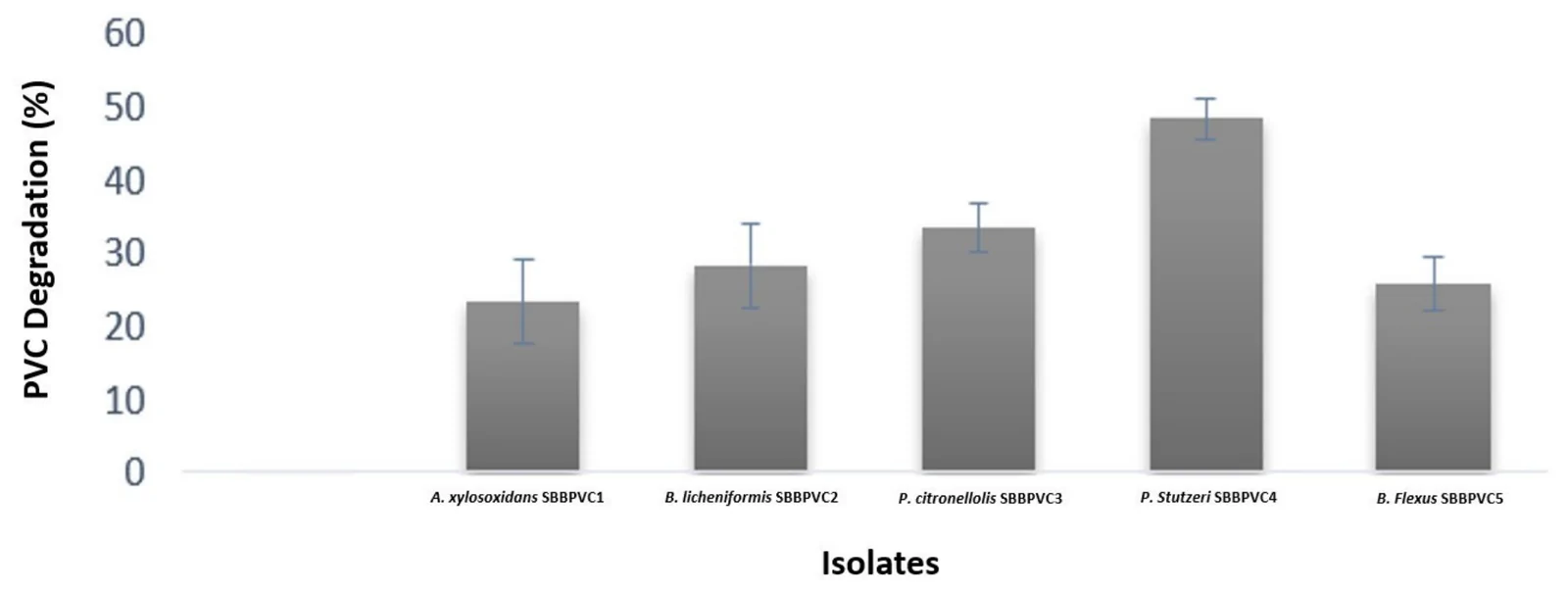
Confirmation of PVC degradation potential of bacteria through weight loss experiment.

The error bar (SD) magnitude in Figure 3 is not uniform across time because replicate variability is growth-phase dependent. During exponential growth (24–30 h), replicate cultures track each other closely and SDs are small across all isolates. Once cultures enter the stationary/decline phase (48–72 h), small differences in the timing of nutrient depletion and cell lysis between replicate flasks cause SD to increase—this is most visible in *flexus*SBBPVC5, whose SD peaks at 54 h (SD = 0.0245, versus ≤0.009 for all other isolates at any time point), coinciding with its earliest and steepest decline in OD_600_ among the five isolates.

Of 45 time-point comparisons (5 isolates × 9 time points), 44 (97.8%) showed significant growth compared to the control (*p* < 0.05). The only non-significant result was *B. flexus* SBBPVC5 at time 0 h (*p* = 0.9781). All isolates demonstrated significant growth by 3 h, post inoculation (*p* < 0.001 for all), indicating rapid adaptation to growth conditions (Supplementary Materials, Table S5).

### 3.7. Confirmation of PVC Degradation via Weight Loss Assay

Optimum PVC degradation (50%) was observed in *P. stutzeri* SBBPVC4. *P. citronellolis* SBBPVC3 (33.33%) and *B. flexus* SBBPVC5 (28.5%) displayed moderate degradation. The least weight loss was observed in *A. xylosoxidans* SBBPVC1 (20%) and *B. licheniformis* SBBPVC2 (25%) (Figure 3). The distinct variations in PVC degradation potential among the isolates can be attributed to differences in their enzymatic repertoires, cell-surface properties and metabolic adaptations.

All five isolates demonstrated statistically significant PVC degradation compared to the 0% control baseline (*p* < 0.01 for all). Isolate-to-isolate comparisons for the PVC degradation assay were performed via one-way ANOVA (F4,10 = 14.81, *p* = 0.0003) followed by Bonferroni-corrected pairwise post hoc tests (10 comparisons, αcorrected = 0.005). These confirmed that *P. stutzeri* SBBPVC4 significantly outperformed all four other isolates (all *p* < 0.01), while the isolates ranked 2–5 did not differ significantly from one another (Table 1).

### 3.8. FTIR Analysis

**Figure 4:**
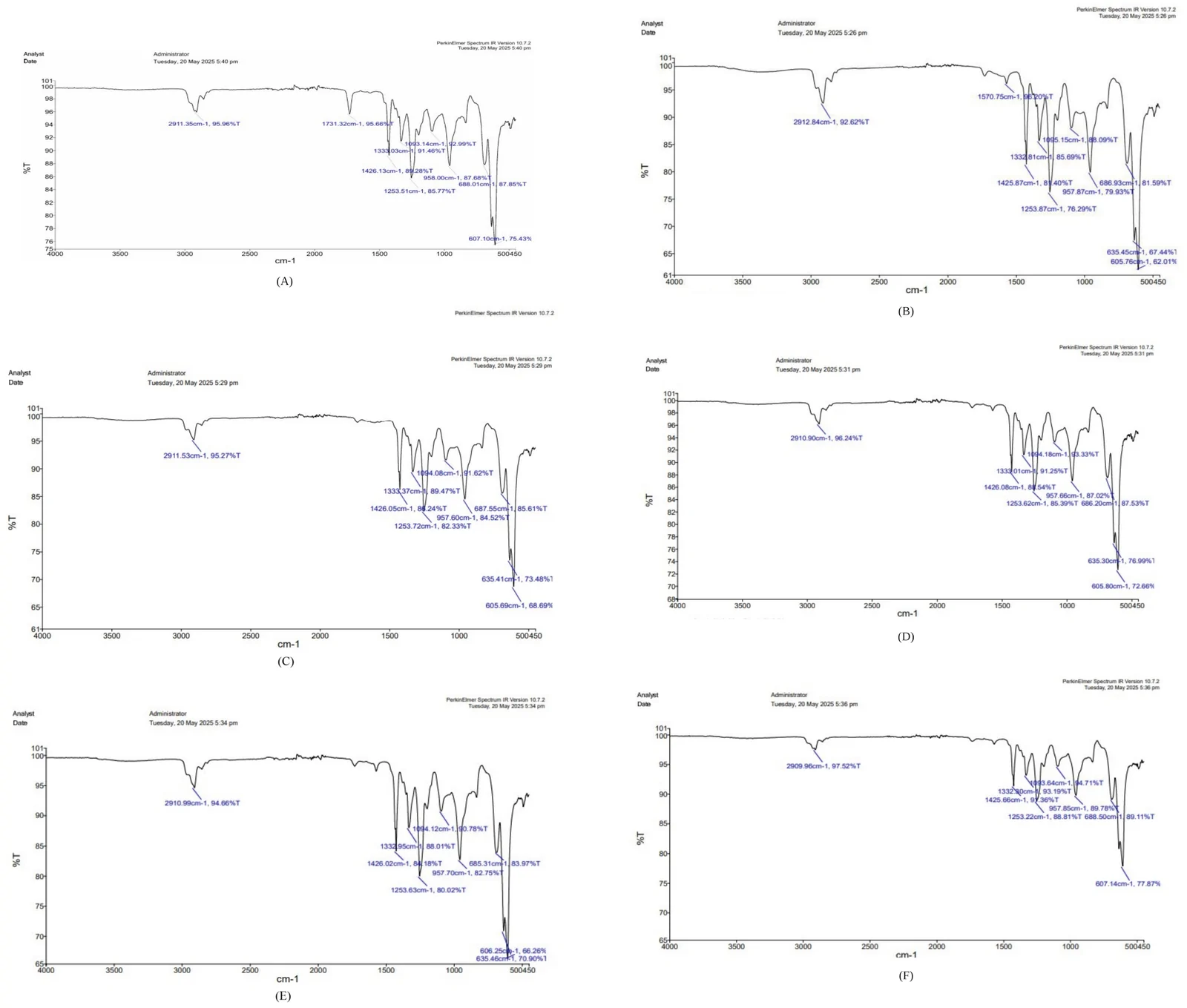
Confirmation of PVC degradation potential of bacteria based on FTIR analysis: (**A**) control (**B**) *A. xylosoxidans* (SBBPVC1); (**C**) *B. licheniformis* (SBBPVC2); (**D**) *P. citronellolis* (SBBPVC3); (**E**) *P. stutzeri* (SBBPVC4); and (**F**) *B. flexus* (SBBPVC5).

FTIR spectra of bacteria-treated PVC strips showed variations in regions of 500–1500 and 2800–3000 cm^−1^, corresponding to -C–H, =C–H and –C–O bonds, respectively (Table 2). The *A. xylosoxidans*-treated strips spectrum demonstrated a reduction of 62% in the C-Cl peak and the appearance of a peak at 1650 cm^−1^, reflecting carbonyl and carboxyl groups formation. It confirmed backbone breakdown via oxidation. Spectra of strips treated with *B. licheniformis* revealed a reduced intensity of peaks in regions of 607 and 1253 cm^−1^. FTIR spectra of *P. citronellolis*-treated strips demonstrated reduction in C-Cl bond stretching and the appearance of new peaks. Similar to the spectra of strips exposed to *B. licheniformis*, *P. stutzeri*-treated PVC spectra showed reduced peaks of intensity in C-O and C-Cl regions (Figure 4).

### 3.9. Confirmation of PVC Degradation via SEM Analysis

**Figure 5:**
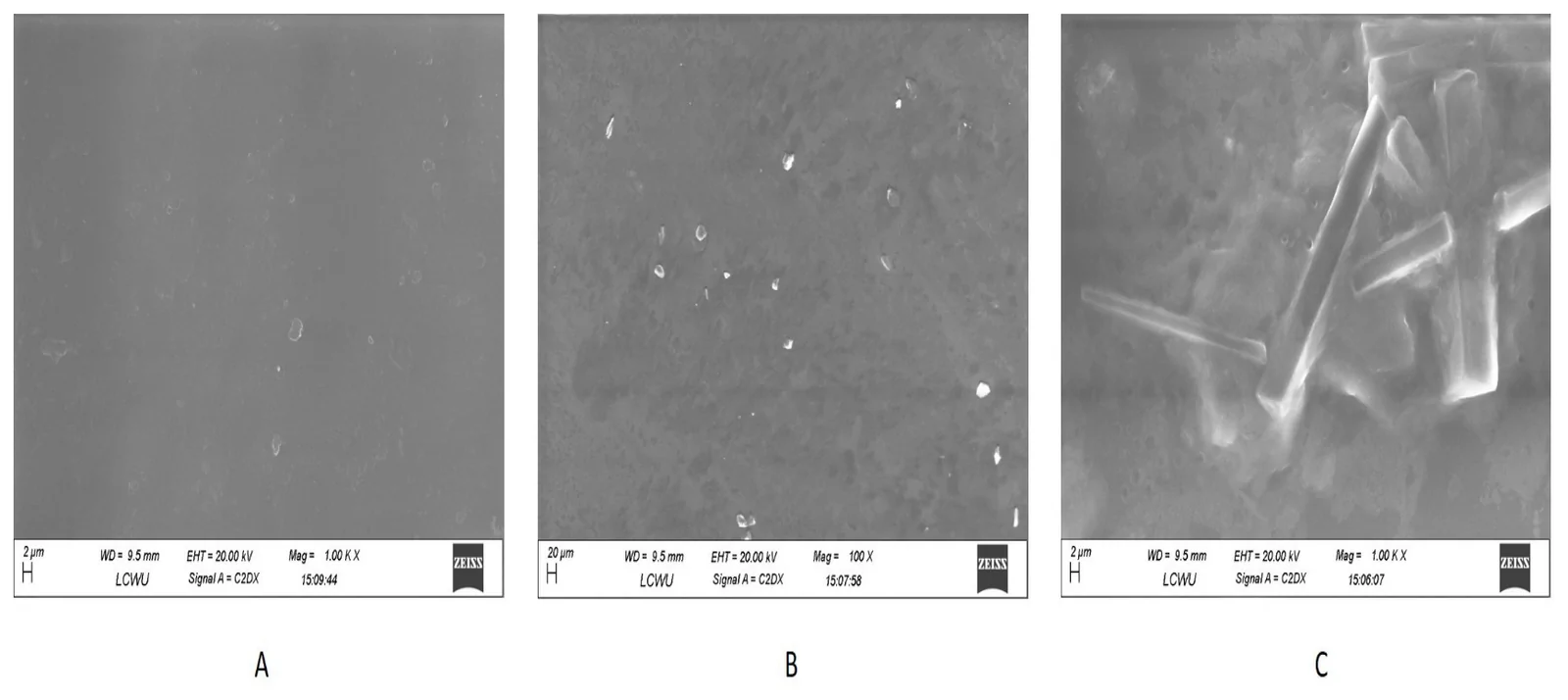
Confirmation of degradation of PVC films by *P. citronellolis* SBBPVC3 based on SEM analysis: (**A**) Abiotic control PVC; (**B**) NaOH-treated PVC; and (**C**) bacteria-treated PVC.

PVC strips treated with NaOH before inoculation demonstrated minor grooves in SEM micrographs. On the other hand, micrographs of strips exposed to *P. citronellolis* SBBPVC3 showed structural fracturing and deep pitting on surfaces, confirming the bacterial degradation (Figure 5).

### 3.10. GC–MS Analysis

Among the five bacterial isolates under study, two isolates were selected (*A. xylosoxidans* SBBPVC1 and *P. citronellolis* SBBPVC3) for GC–MS metabolic profiling based on efficient PVC breakdown. The resulting spectra were analyzed using the NIST library and through a comparison of *m*/*z* ratios and fragmentation patterns of GC–MS spectra obtained in the current study with *m*/*z* ratios and fragmentation patterns of the PVC degradation compounds reported in the literature. The NIST Library enabled the identification of eight compounds in *A. xylosoxidans* SBBPVC1, including 3,4-dihydroxymandelic acid, 2,4-dihydroxybenzoic acid, phthalic acid, di(2-propylpentyl) ester, 1,2-benzenediol, 3,5-bis(1,1-dimethylethyl)-, anthracene, 9,10-dihydro-9,9,10-trimethyl-, benzo[h]quinoline, 2,4-dimethyl-, anthracene, 9,10-diethyl-9,10-dihydro- and benzo[h]quinoline, 2,4-dimethyl (Table 3).

**Figure 6:**
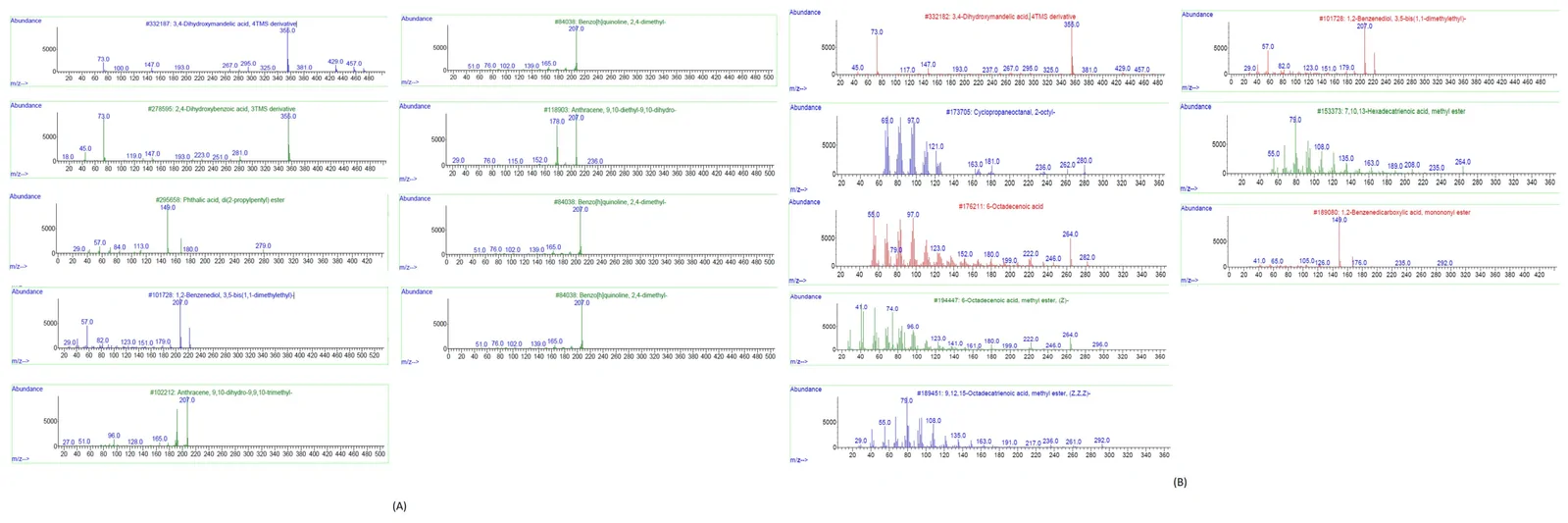
PVC degradation metabolites identified in (**A**) *A. xylosoxidans* SBBPVC1 and (**B**) *P. citronellolis* SBBPVC3, based on GC-MS analysis via comparison with NIST Library.

Comparison of GC–MS spectra with the NIST Library revealed eight compounds in *P. citronellolis* SBBPVC3, i.e., cyclopropaneoctanal, 3,4-dihydroxymandelic acid, 2-octyl-, 6-octadecenoic acid, 1,2-benzenedicarboxylic acid, monononyl ester, 6-octadecenoic acid, methyl ester, (z)-, 9,12,15-octadecatrienoic acid, methyl ester, (z,z,z)-, 7,10,13-hexadecatrienoic acid, methyl ester and 1,2-benzenediol, 3,5-bis(1,1-dimethylethyl) (Table 4 and Figure 6).

The comparison of GC–MS spectral *m*/*z* ratios with fragmentation patterns of PVC degradation intermediate compounds documented in the literature showed the identification of aliphatic aldehydes and alcohols, esters, including 1-methyl heptyl, isooctyl, adipic acid, methyl octyl and octyl myristate esters, and citrate, isocitrate, alpha ketoglutarate, succinyl-CoA, succinate and malate (Table 5).

### 3.11. Antibiotic Sensitivity Profiling

**Figure 7:**
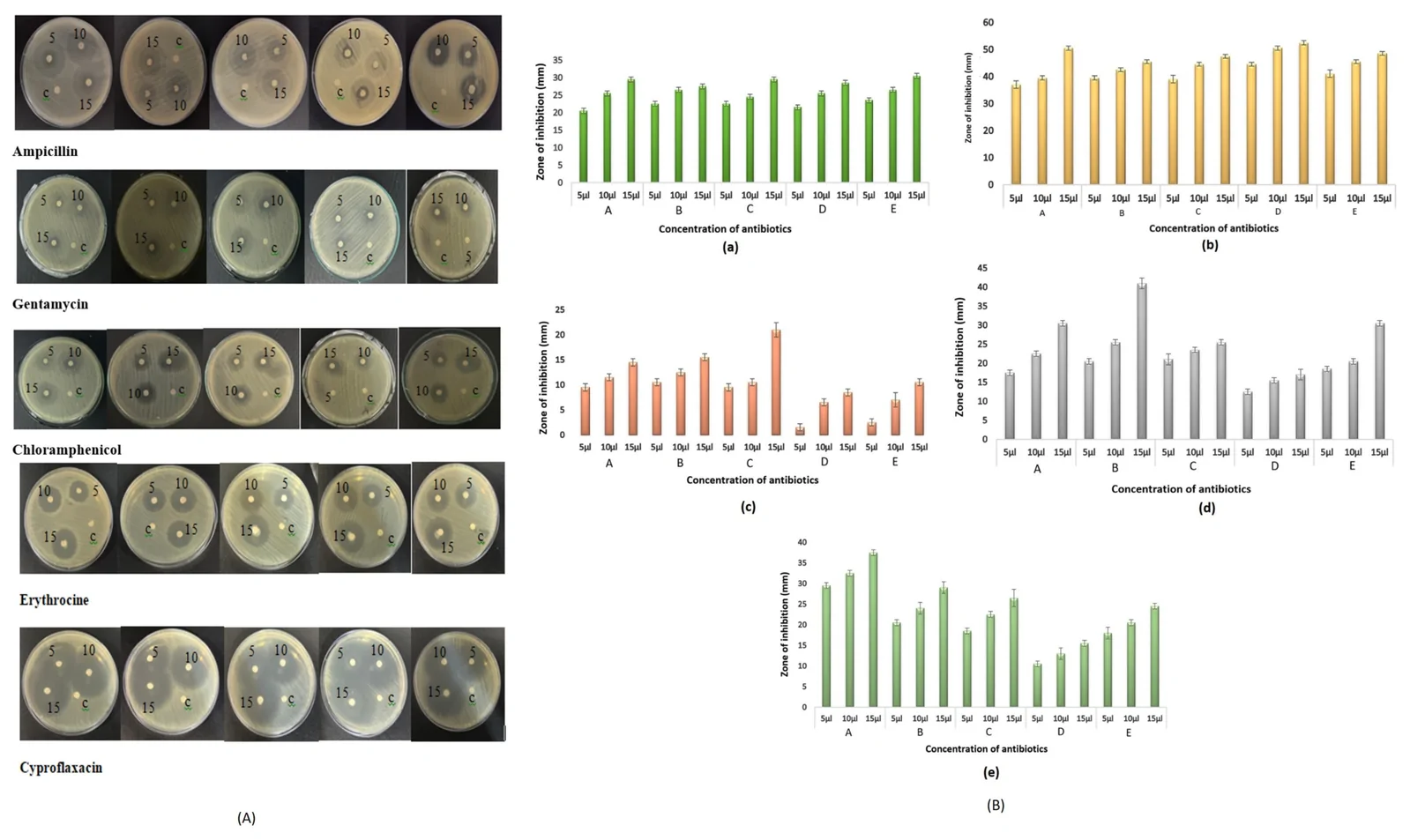
Antibiotic sensitivity profiling of PVC degrading bacteria [43]: (**A**) zones of inhibition exhibited by five bacterial strains in response to varying concentrations (5, 10 and 15 µL) of erythrocin, ciprofloxacin, gentamycin, chloramphenicol, ampicillin, c with green wave line is showing the control (**B**) graphical presentation of antibiotic resistance potential of bacteria, (**a**) *A. xylosoxidans* SBBPVC1, (**b**) *B. licheniformis* SBBPVC2; (**c**) *P. citronellolis* SBBPVC3; (**d**) P. *stutzeri* SBBPVC4; and (**e**) *B. flexus* SBBPVC5, Labels along x-axis: A: erythromycin, B: ciprofloxacin, C: chloramphenicol, D: gentamycin, E: ampicillin.

Optimum susceptibility was observed in isolates against ciprofloxacin. Strong susceptibility was demonstrated in *P. stutzeri* SBBPVC4, whereas isolate *A. xylosoxidans* SBBPVC1 showed rising zones with increasing concentrations. *A. xylosoxidans* SBBPVC1 and *B. licheniformis* SBBPVC2 exhibited large inhibition zones at the maximum concentration of gentamicin (Figure 7). *P. stutzeri* SBBPVC4 showed especially poor sensitivity, while most isolates showed tiny inhibitory zones against chloramphenicol. Generally, ampicillin was consistently effective, except for *A. xylosoxidans* SBBPVC1, which displayed a comparatively higher susceptibility. Only mild susceptibility was observed in other isolates.

Of the 75 tests performed (5 isolates × 5 antibiotics × 3 doses), 73 (97.3%) demonstrated statistically significant inhibition (*p* < 0.05) compared to the zero-inhibition control. Two non-significant results were observed, both involving chloramphenicol at the 5 µL dose: *P. stutzeri* SBBPVC4 (*p* = 0.1024) and *B. flexus* SBBPVC5 (*p* = 0.0628) (Supplementary Materials, Table S6). The antibiotic sensitivity assay used n = 2 technical replicates per condition, which produces very wide 95% CIs (t-critical at df = 1 is 12.706). The CI is a poor summary of precision; the one-sample *t* test *p*-value remains the primary basis for the significance calls in that assay. Significant/non-significant language refers strictly to the statistical comparison against the zero-inhibition baseline and reserves the terms “susceptible/resistant” for describing the biological pattern (presence/absence of a meaningful zone) to avoid implying formal clinical breakpoint categorization.

## 4. Discussion

The five PVC-degrading isolates identified in current study were: *A. xylosoxidans*, *B. licheniformis*, *P. citronellolis*, *P. stutzeri*, *B. toyonensis* and *B. flexus.* This finding is consistent with previous work, as various studies have documented bacteria belonging to these genera and species [13,44,45,46,47,48,49].

IAA and HCN production are ecological fitness traits. IAA helps the bacteria to colonize plant roots near buried plastics. HCN acts as an antifungal/antibacterial weapon, giving a PVC-degrading strain a competitive advantage to dominate the biofilm on the plastic surface.

The observed growth patterns revealed distinct metabolic strategies: *B. licheniformis* SBBPVC2 prioritized biomass accumulation with sustained high-density growth, while *P. stutzeri* SBBPVC4 employed a rapid-growth strategy with a faster metabolic rate but lower final biomass. These differences may correlate with degradation efficiency, as rapid metabolism could facilitate enzymatic degradation of recalcitrant substrates like PVC.

The observed growth kinetics showed strain-specific alterations in PVC degradation potentials. *B. flexus* SBBPVC5 and *P. stutzeri* SBBPVC4 demonstrated rapid initial growth in a PVC-enriched environment [50]. *B. licheniformis* SBBPVC2 and *P. citronellolis* SBBPVC3 exhibited logarithmic growth during 6–24 h, reflecting their enzymatic potential for PVC degradation [51]. Notably, *B. licheniformis* exhibited a prolonged stationary phase and delayed death phase, suggesting that sustained metabolism and enzyme production make it a promising option for long-term bioremediation [52].

All isolates exhibited significant PVC biodegradation, i.e., 20–50%/30 days. *P. stutzeri* is known for its high cell-surface hydrophobicity and production of enzymes such as esterases, lipases and oxygenases. The lower efficiency of *P. citronellolis* SBBPVC3 and *B. flexus* SBBPVC5, as compared to *P. stutzeri*, may stem from lower enzyme expression levels or less efficient binding affinity to the hydrophobic plastic surface. The limited weight loss induced by *A. xylosoxidans* SBBPVC1 and *B. licheniformis* SBBPVC2 suggests that their enzymatic machinery may lack the specific depolymerases required to breach the highly crystalline regions of PVC.

Contrary to the current study, several bacteria documented in the literature showed less efficient degradation potential, i.e., *Altermonas* BP-4.3 (1.76%/60 days), *Bacillus licheniformis* Sb.1 and *A. xylosoxidans* Sb. 2 (15 and 17%/30 days) and *Bacillus* (12.7%/6 months) and *Micrococcus* (8.4%/6 months) [6,12,53]. *A. xylosoxidans* showed 20% PVC weight loss, aligning with the reported degradation ranges (20%/2 weeks) for *Pseudomonas* and *Brevibacterium* [54].

Contrary to previous published work, *P. stutzeri* SBBPVC4 demonstrated exceptional PVC bioremediation potential, degrading nearly half of the substrate mass under experimental conditions. The 48.33% degradation efficiency substantially exceeds values reported in many published PVC degradation studies, suggesting that this isolate harbors potent enzymatic machinery for polymer breakdown. The correlation between the rapid growth rate (0.0683 OD^600^/hour) and high degradation efficiency suggests that metabolic activity is a key driver of PVC degradation capacity.

Pure, untreated PVC is highly hydrophobic, chemically inert, and resistant to microbial colonization due to its high chlorine content. In a weight loss assay, pre-treatment with NaOH served two core functions, i.e., it initiated PVC de-chlorination and de-plasticization, significantly enhancing subsequent microbial degradation and weight [55,56]. Pre-treated PVC microplastics typically exhibit notable weight loss acceleration when exposed to degrading strains, often yielding considerable gravimetric weight losses as compared to negligible losses (<1%) in untreated controls [45,57].

*P. stutzeri* SBBPVC4 exhibited the highest gravimetric weight loss, likely due to rapid initial biomass accumulation or non-specific binding to the extracellular matrix. Although all isolates underwent metabolite extraction, they yielded negligible metabolic extracts, with the exceptions of *A. xylosoxidans* SBBPVC1 and *P. citronellolis* SBBPVC3. This suggests that *P. stutzeri* SBBPVC4 may rapidly mineralize substrates into carbon dioxide (CO_2_) without accumulating stable intermediates, rendering GC–MS ineffective for capturing its metabolic fingerprint. Consequently, only *A. xylosoxidans* SBBPVC1 and *P. citronellolis* SBBPVC3 were subjected to targeted metabolic profiling via GC–MS and structural analysis, using FTIR to elucidate specific enzymatic breakdown pathways and functional group modifications.

*P. stutzeri* SBBPVC4 is known for its high cell-surface hydrophobicity and the production of robust extracellular enzymes such as esterases, lipases and oxygenases. These enzymes effectively break down the resilient carbon–carbon backbone and plasticizer bonds of PVC. Furthermore, its ability to form dense biofilms allows for prolonged, localized enzymatic attack on the polymer surface. *P. citronellolis* SBBPVC3 and *B. flexus* SBBPVC5 exhibited moderate degradation because they likely possess the necessary metabolic pathways to utilize PVC additives or intermediate degradation products as carbon sources. However, their lower efficiency compared to *P. stutzeri* may stem from lower enzyme expression levels or less efficient binding affinity to the hydrophobic plastic surface. *A. xylosoxidans* SBBPVC1 and *B. licheniformis* SBBPVC2 demonstrated the lowest amount of PVC degradation because their enzymatic machinery may lack the specific depolymerases required to breach the highly crystalline regions of PVC, limiting their activity to superficial degradation.

FTIR analysis identified strain-specific PVC breakdown, with *A. xylosoxidans* SBBPVC1 demonstrating significant changes in spectra such as the reduction in C–Cl bond peaks and the appearance of a carbonyl peak, indicating oxidation of the PVC carbon chain. This finding is in line with studies in the literature [58]. Moderate degradation was observed in the case of *B. licheniformis* and *P. stutzeri*, based on C–Cl and C–O peaks reduction in FTIR spectra [36]. They target PVC plasticizers (e.g., phthalates) rather than the PVC backbone, contributing to major weight loss. The negligible spectral modifications corresponding to surface erosion observed in the spectra of PVC strips treated with *B. flexus* and *P. citronellolis* is also consistent with previous studies in the literature [45].

Despite the fact that PVC exhibits exceptional recalcitrance due to non-hydrolysable ester bonds, current study bacteria achieved 50% weight loss in the PVC strips per 30 days. The synergistic action of alkali and bacteria contributed to this weight loss. Alkali treatment introduces carbonyl (C=O) and hydroxyl (-OH) groups and unsaturated double bonds in PVC, thereby increasing its hydrophilicity. These functional groups and double bonds are easily targeted by bacteria for carbon-chain degradation.

**Figure 8:**
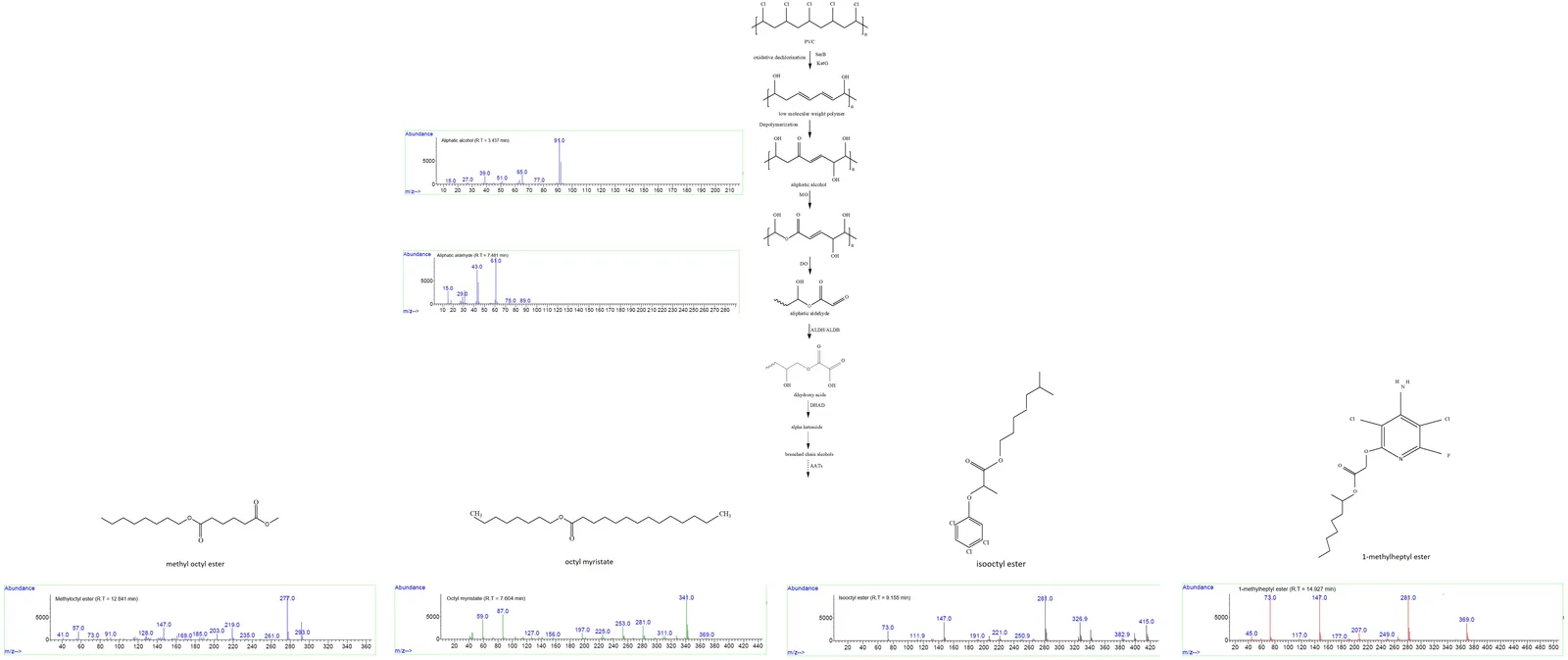
PVC degradation pathway hypothesized in bacteria through GC–MS-based metabolic profiling [59,60,61,33,34]. SerB: dehalogenase, KatG: catalase peroxidase, MO: monooxygenase, DO: dioxygenase, ALDH: aldehyde dehydrogenase, ALDB: aldehyde dehydrogenase B, DHAD: dihydroxyacid dehydrogenase, AATs: alcohol acyltransferases.

Based on the GC–MS analysis, the presence of the earlier documented pathway of PVC degradation is hypothesized in *A. xylosoxidans* SBBPVC1 and *P. citronellolis* SBBPVC3. In this pathway, initially, PVC undergoes oxidation and dechlorination in the presence of the enzymes dehalogenase (SerB) and catalase peroxidase (KatG), respectively. It leads to the formation of lower molecular-weight polymers. Following this, the oxidation of polymers occurs in the presence of monooxygenases (MO), dioxygenases (DO), laccases, esterases (ESTs) and dehydrogenases, leading to the production of carbonyl and hydroxyl compounds. These compounds are then transformed into fatty acids esters like adipic acid, methyl-octyl ester, octyl myristate, isooctyl ester and 1-methylheptyl ester (Figure 8).

The conversion of PVC into lower molecular-weight polymers has also been reported in previous work on *Burkholderia*, *Dyella* and *Rhodococcus* [57]. The formation of carbonyl and hydroxyl compounds is also in line with studies in the literature, as aliphatic alcohols and aldehydes formation were observed in *Klebsiella* sp. EMBL-1 [58]. Esterification of aliphatic alcohols and aldehydes is also supported by earlier work documenting the formation of 1-methyl heptyl, isooctyl, methyl octyl and octyl myristate esters. However, contrary to this study, a few esters, like 2-nonanol, adipic acid, dioctyl adipate (DOA) and dioctyl terephthalate (DOTP), were not identified in PVC bacteria [36].

Weight loss, FTIR, GC–MS and SEM analysis of *P. citronellolis* SBBPVC3 demonstrated PVC chemical modifications leading to low molecular-weight fragment formation. However, we could not confirm PVC backbone degradation among all bacteria, as GPC-based molecular-weight distribution and SEM-based surface topography changes were not documented. As a consequence, the PVC structural changes observed should be considered as primary evidence of degradation. Elucidation of backbone-degrading pathways via the tracking of molecular-weight shifts will be focused on in future research.

In the antibiotic sensitivity assays, we did not include a solvent-only control. However, the contribution of the carrier solvent to the observed zones cannot be formally excluded, and we recommend a solvent-only control be included in future work with these isolates.

Antibiotic susceptibility profiles reflect bacterial virulence. Since resistant strains can spread multidrug resistance (MDR), using live cells for bioremediation poses significant risks. Rather than utilizing whole organisms, we can clone their genes into safe expression hosts or synthesize enzyme-based nanoparticles to remediate PVC in real-world scenarios.

## 5. Conclusions

The current study found that *P. stutzeri* SBBPVC4 is the optimum PVC degrader, with 48.33% degradation and the fastest growth rate, i.e., 0.0683 OD^600^/hour. This bacterium significantly outperformed other isolates. The proposed mechanisms of PVC degradation suggested in this study are hypothesized pending validation at the genetic and enzymatic levels. The isolation of PVC-degrading bacteria, achieving an efficiency of 20 to 50% per 30 days, marks a significant milestone in overcoming the persistent resilience of PVC. While optimizing this microbial process for industrial application will entail further insights into environmental variables and genomics, transcriptomic and proteomics analysis confirmed specific enzymatic pathways. This will establish a practical scheme for bio-based modern plastic recycling systems.

## Figures and Tables

**Table 1:** Statistical analysis of PVC weight loss assay via one-sample *t* test and ANOVA and post hoc ranking.

Isolate	Mean_Degradation (%)	SD (%)	t-Statistic	95% (CI) %	*p*-Value	Significance
Control	0	0	---	----	<0.0001	ns
*A. xylosoxidans* SBBPVC1	23.33	5.77	7	9.00–37.66	0.0099	**
*B. licheniformis* SBBPVC2	28.33	5.77	8.5	14.00–42.66	0.0068	**
*P. citronellolis* SBBPVC3	33.33	3.34	17.31	25.03–41.63	0.0017	**
*P. stutzeri* SBBPVC4	48.33	2.89	29	41.15–55.51	0.0006	***
*B. flexus* SBBPVC5	25.71	3.78	11.79	16.32–35.10	0.0036	**

*p* < 0.001 (***), *p* < 0.01 (**).

**Table 2:** Confirmation of PVC strips degradation by current study in documented bacteria based on FTIR analysis.

Organism	C–Cl (607 cm^−1^)	1253 cm^−1^ (C–O)	Other Key Shifts	Interpretation
*A. xylosoxidans* (SBBPVC1)	↓ 62.0%T	↓ 76.3%T	New peak at 1570.7 cm^−1^	Highest degradation, oxidative changes
*B. licheniformis* (SBBPVC2)	↓ 68.7%T	↓ 82.3%T	Slight peak weakening across region	Moderate degradation, evident chain attack
*P. citronellolis* (SBBPVC3)	↓ 72.6%T	85.4%T (close to CA)	Minor changes	Mild degradation, early-stage alteration
*P. stutzeri* (SBBPVC4)	↓ 66.2%T	↓ 80.0%T	Slight peak shift at 685–688 cm^−1^	Moderate, closer to P2
*B. flexus* (SBBPVC5)	77.9%T (near CA)	88.8%T	Nearly same as control	Negligible degradation

**Table 3:** Metabolites of PVC degradation identified in *A. xylosoxidans* SBBPVC1-based comparison of GC–MS spectra with spectra of NIST Library compounds.

No	Compound Name	Mol wt. (g/mol)	Retention Times (min)	*m*/*z*	NIST Library Match Score	Reference
1	3,4-Dihydroxymandelic acid	184.15	11.499	73, 147, 184, 267, 295, 355, 381, 429, 457	87	[33]
2	2,4-Dihydroxybenzoic acid	154.12	13.408	45, 73, 147, 154, 223, 251, 281, 355	95%	[34]
3	Phthalic acid, di(2-propylpentyl) ester	390.56	16.285	57, 84, 113, 149, 279, 390	85	[35]
4	1,2-Benzenediol, 3,5-bis(1,1-dimethylethyl)-	222.32	16.847	29, 57, 82, 123, 151, 179, 207, 222	90%	[36]
5	Anthracene, 9,10-dihydro-9,9,10-trimethyl-	222.33	18.403	51, 96, 165, 207, 222	87%	[37]
6	Benzo[h]quinoline, 2,4-dimethyl-	207.27	18.500	76, 102, 139, 165, 207	86%	[38]
7	Anthracene, 9,10-diethyl-9,10-dihydro-	236.35	18.805	29, 76, 115, 152, 178, 207, 236	88%	[39]
8	Benzo[h]quinoline, 2,4-dimethyl-	297.27	18.965	51, 76, 102, 139, 165, 207	85%	[38]

**Table 4:** Metabolites of PVC degradation identified in *P. citronellolis* SBBPVC3 based on comparison of GC–MS spectra with spectra of NIST Library compounds.

No	Compound Name	Mol. wt. (g/mol)	*m*/*z*	Retention Time (min)	NIST Library Match Score	Reference
1	3,4-Dihydroxymandelic acid	184.15	45, 73, 147, 184 193, 267, 295, 355	13.408	95%	[33]
2	Cyclopropaneoctanal, 2-octyl-	280.5	69, 97, 121, 163, 181, 236, 262, 280	13.836	85%	[40]
3	6-Octadecenoic acid	282.46	55, 79, 97, 123, 152, 180, 199, 222, 246, 264, 282	13.836	90%	[36]
4	6-Octadecenoic acid, methyl ester, (Z)-	296.49	41, 74, 96, 123, 141, 161, 180, 222, 246, 264, 296	13.880	89%	[40]
5	9,12,15-Octadecatrienoic acid, methyl ester, (Z,Z,Z)-	292.46	29, 55, 79, 108, 135, 163, 191, 217, 236, 261, 292	13.873	88%	[39]
6	7,10,13-Hexadecatrienoic acid, methyl ester	264.40	55, 79, 108, 135, 163, 189, 208, 235, 264	13.873	93%	[41]
7	1,2-Benzenedicarboxylic acid, monononyl ester	292.37	41, 65, 126,149, 176, 235, 292	16.285	90%	[39]
8	1,2-Benzenediol, 3,5-bis(1,1-dimethylethyl)-	222.32	29, 57, 82, 123, 151, 179, 207, 222	18.307	85%	[42]

**Table 5:** Metabolites and pathways identified in PVC-metabolizing bacteria through comparison of *m*/*z* ratios of current study bacterial GC–MS spectra with fragmentation patterns of earlier reported PVC-degrading bacterial metabolites.

Metabolite Identified	SBBPVC1	SBBPVC3	Chemical Formula	Fragmentation Ion	Molecular wt. g/mol	FTIR Spectral Peaks (cm^−1^)	Retention Time
Aliphatic aldehyde	I	I	C_n_H_2n_O	29, 41, 44, 58, 72	variable	1720–1740 (C=O stretch) 1350–1470 (C-H bending)	7.481
Aliphatic alcohol	I	I	C_n_H_2n+1_OH	18, 31, 42, 45, 59, 73	variable	1000–1100 (C-O stretch)	3.437
1-methyl heptyl ester	I	I	C_15_H_21_FN_2_O_3_	43, 61, 112, 367	367.24	1720–1740 (C=O stretch) 1100–1250 (C-O stretch) 1460 (CH_2_ bending) 1370 (CH_3_ bending)	14.927, 18.805, 7.706, 14.927
Isooctyl ester	I	I	C_17_H_23_Cl_3_O_3_	56, 70, 84, 98, 112, 149, 381	381.7259		9.155, 10.439,
Adipic acid, methyl octyl ester	I	I	C_15_H_28_O_4_	43, 59, 57, 71, 74, 87, 101, 115, 143, 272	272.38		9.155, 19.339, 12.841,
Octyl myristate	I	I	C_22_H_44_O_2_	43, 57, 112, 228, 340	340.592		7.604, 7.706, 14.199, 14.927, 18.500,
Citrate	I	I	C_6_H_5_O_7_^3−^	41, 67, 110, 190, 85, 133	189.09	1570 cm^−1^ (Coo^−^ Asym Stretch), 1425 cm^−1^ (Coo^−^ Sym Stretch), 1253 cm^−1^ (C–O)	18.306, 15.612
Isocitrate	I	I	C_6_H_8_O_7_	73, 85, 173, 347, 147	192	1570 cm^−1^ (Coo^−^ Stretch), 1094 cm^−1^ (C–O Stretch)	20.434, 14.200
α-ketoglutarate	I	I	C_5_H_6_O_5_	73, 75, 147, 56, 74	146	1570 cm^−1^ (Coo^−^ Asym Stretch), 1700 cm^−1^ (C=O Stretch)	18.855, 13.406
Succinyl-CoA	I	_	C_25_H_40_N_7_O_19_P_3_S	361, 429, 135, 361, 460	867	1570 cm^−1^ (Coo^−^ Stretch), 1735 cm^−1^ (C=O Thioester Stretch)	18.457
Succinate	I	_	C_4_H_4_O_4_^2−^	73, 147, 261, 235	116	1570 cm^−1^ (Coo^−^ Stretch), 1425 cm^−1^ (Coo^−^ Sym Stretch)	12.842
Malate	I	_	C_4_H_4_O_5_	71, 117, 29, 43, 89	132	1570 cm^−1^ (Coo^−^ Stretch), 1094 cm^−1^ (C–O Stretch), 1425 cm^−1^ (Ch_2_/Coo^−^)	18.903

## Data Availability

The FASTA files have been submitted to NCBI under the accession numbers PV794637, PV797396, PV797336, PV797410 and PV797561.

## Supplementary Materials

The following supporting information can be downloaded at: https://www.mdpi.com/article/10.3390/microorganisms14092114/s1, Table S1: Colony forming units (CFUs) enumeration of the current study bacteria at different dilution factors. Table S2: Morphological characterization of PVC-degrading isolates. Table S3: Absorbance values of bacterial isolates at 600 (nm) over 72 h’ period. Table S4: Growth phases of PVC degrading bacterial isolates measured based on OD^600^. Table S5: Statistical analysis of growth curve study via time-point-by-time-point comparison and growth parameter extraction. Table S6: Statistical analysis of antibiotic sensitivity profiling of PVC degrading isolates via One-Sample T-test. Figure S1: Sites in Multan selected for soil sample collection for isolation of PVC degrading bacteria (A and B) and Cling foil used as PVC substrate for current study documented bacteria (C). Figure S2: CFUs of PVC-degrading bacteria isolated by the serial dilution method. Figure S3: Biochemical characterization of current study bacteria via indole acetic acid (IAA) production and glucose (Glu), sucrose (SUC) and lactose (LAC) fermentation tests. Figure S4: Biochemical characterization of current study bacteria via catalase (CAT), mannitol sorbitol agar (MSA) and hydrogen cyanide (HCN) production tests. Figure S5: Agarose gel electrophoresis showing (A) extracted genomic DNA and (B) PCR amplicons of current study PVC degrading bacteria.

## References

[1] Pignattelli S., Broccoli A., Piccardo M., Terlizzi A., Renzi M. (2021). Effects of polyethylene terephthalate (PET) microplastics and acid rain on physiology and growth of *Lepidium sativum*. Environ. Pollut..

[2] Olam M. (2023). Mechanical and thermal properties of HDPE/PET microplastics, applications, and impact on environment and life. Advances and Challenges in Microplastics.

[3] Ahmad I., Ali A., Shabbir K. (2025). Plastic pollution, treatment and management strategies in Pakistan: A review. Combating Plastic Pollution in Terrestrial Environment: Challenges and Strategies for a Sustainable Future.

[4] Ziad M., Khan S., Miandad R., Ali G., Hashmi M.Z., Ahmed Z. (2021). Assessment of plastic waste generation and its feasibility for establishment of plastic waste refinery. Arab. J. Geosci..

[5] Akhtar A.S. (2022). The Struggle for Hegemony in Pakistan: Fear, Desire and Revolutionary Horizons.

[6] Saeed S., Iqbal A., Deeba F. (2022). Biodegradation study of polyethylene and PVC using naturally occurring plastic degrading microbes. Arch. Microbiol..

[7] Ali M.I., Ahmed S., Robson G., Javed I., Ali N., Atiq N., Hameed A. (2014). Isolation and molecular characterization of polyvinyl chloride (PVC) plastic degrading fungal isolates. J. Basic Microbial..

[8] Bueno-Ferrer C., Garrigós M.C., Jiménez A. (2010). Characterization and thermal stability of poly (vinyl chloride) plasticized with epoxidized soybean oil for food packaging. Polym. Degrad. Stab..

[9] Miliute-Plepiene J., Fråne A., Almasi A.M. (2021). Overview of polyvinyl chloride (PVC) waste management practices in the Nordic countries. Clean. Eng. Technol..

[10] Wojnowska-Baryła I., Bernat K., Zaborowska K.M. (2022). Plastic waste degradation in landfill conditions: The problem with microplastics, and their direct and indirect environmental effects. Int. J. Environ. Res. Public Health.

[11] Andrady A.L., Heikkilä A.M., Pandey K.K., Bruckman L.S., White C.C., Zhu M., Zhu L. (2023). Effects of UV radiation on natural and synthetic materials. Photochem. Photobiol. Sci..

[12] Khandare S.D., Chaudhary D.R., Jha B. (2021). Bioremediation of polyvinyl chloride (PVC) films by marine bacteria. Mar. Pollut. Bull..

[13] Možar K.B., Miloloža M., Martinjak V., Cvetnić M., Bulatović V.O., Mandić V., Bafti A., Ukić Š., Grgić D.K., Bolanča T. (2023). Bacteria and yeasts isolated from the environment in biodegradation of PS and PVC microplastics: Screening and treatment optimization. Environments.

[14] Kumar B.S.K., Chari N.V.H.K., Reddy K.K., Cheriyan E., Sherin C.K., Rao D.B., Elangovan S.S., Reddy B.B., Gupta G.V.M. (2024). Natural light driven plastic leaching effects on carbon chemistry in the tropical coastal waters of eastern Arabian sea: An experimental study. Environ. Pollut..

[15] Khandare S.D., Chaudhary D.R., Jha B. (2025). Isolation and purification of esterase enzyme from marine bacteria associated with biodegradation of polyvinyl chloride (PVC). Biodegradation.

[16] Naveed M., Naveed R., Aziz T., Azeem A., Afzal M., Waseem M., Alherbi M., Alshammari A., Alasmari A.F., Albekairi T.H. (2024). Biodegradation of PVCs through in-vitro identification of Bacillus albus and computational pathway analysis of ABH enzyme. Biodegradation.

[17] Othman A.R., Hasan H.A., Muhamad M.H., Ismail N.I., Abdullah S.R.S. (2021). Microbial degradation of microplastics by enzymatic processes: A review. Environ. Chem. Lett..

[18] Barth M., Oeser T., Wei R., Then J., Schmidt J., Zimmermann W. (2015). Effect of hydrolysis products on the enzymatic degradation of polyethylene terephthalate nanoparticles by a polyester hydrolase from *Thermobifida fusca*. Biochem. Eng. J..

[19] Ameen F., Al-Shwaiman H.A., Almalki R., Al-Sabri A.E., Sholkamy E.N. (2025). Degradation of polyvinyl chloride (PVC) microplastics employing the actinobacterial strain *Streptomyces gobitricini*. Biodegradation.

[20] Rojas I.O., Pérez A.R., González J.F.C., Juárez V.M.M., Domínguez E.E., Oviedo J.T., Rodriguez I.A. (2021). Biodegradation capacity and activity enzymatic of Bacillus subtilis against low-density polyethylene. Asian J. Res. Biochem..

[21] Paray A.A., Singh M., Mir M.A., Kaur A. (2023). Gram staining: A brief review. Int. J. Res. Rev..

[22] El-Ashram S., Al Nasr I., Suo X. (2016). Nucleic acid protocols: Extraction and optimization. Biotechnol. Rep..

[23] Hussain N., Mohiuddin F., Muccee F., Bunny S.M., Al Haddad A.H. (2025). Isolation, molecular, and metabolic profiling of benzene-remediating bacteria inhabiting the tannery industry soil. Pol. J. Microbiol..

[24] Kent W.J. (2002). BLAT—The BLAST-like alignment tool. Genome Res..

[25] Benson D.A., Karsch-Mizrachi I., Lipman D.J., Ostell J., Wheeler D.L. (2005). GenBank. Nucleic Acids Res..

[26] Sievers F., Higgins D.G. (2018). Clustal Omega for making accurate alignments of many protein sequences. Protein Sci..

[27] Tamura K., Stecher G., Kumar S. (2021). MEGA11: Molecular evolutionary genetics analysis version 11. Mol. Biol. Evol..

[28] Evans J., Sheneman L., Foster J. (2006). Relaxed neighbor joining: A fast distance-based phylogenetic tree construction method. J. Mol. Evol..

[29] Seong H.J., Kim H., Ko Y.J., Yao Z., Baek S.B., Kim N.J., Jang Y.S. (2024). Enhancing polyethylene degradation: A novel bioprocess approach using *Acinetobacter nosocomialis* pseudo-resting cells. Appl. Microbiol. Biotechnol..

[30] Sapcariu S.C., Kanashova T., Weindl D., Ghelfi J., Dittmar G., Hiller K. (2014). Simultaneous extraction of proteins and metabolites from cells in culture. MethodsX.

[31] Allen F., Pon A., Greiner R., Wishart D. (2016). Computational prediction of electron ionization mass spectra to assist in GC/MS compound identification. Anal. Chem..

[32] Valdez C.A., Leif R.N., Hok S., Alcaraz A. (2018). Assessing the reliability of the NIST library during routine GC-MS analyses: Structure and spectral data corroboration for 5, 5-diphenyl-1, 3-dioxolan-4-one during a recent OPCW proficiency test. J. Mass Spectrom..

[33] Lv S., Li Y., Zhao S., Shao Z. (2024). Biodegradation of typical plastics: From microbial diversity to metabolic mechanisms. Int. J. Mol. Sci..

[34] Zhang Z., Peng H., Yang D., Zhang G., Zhang J., Ju F. (2022). Polyvinyl chloride degradation by a bacterium isolated from the gut of insect larvae. Nat. Commun..

[35] Kudzin M.H., Piwowarska D., Festinger N., Chruściel J.J. (2023). Risks associated with the presence of polyvinyl chloride in the environment and methods for its disposal and utilization. Materials.

[36] Kotova I.B., Taktarova Y.V., Tsavkelova E.A., Egorova M.A., Bubnov I.A., Malakhova D.V., Shirinkina L.I., Sokolova T.G., Bonch-Osmolovskaya E.A. (2021). Microbial degradation of plastics and approaches to make it more efficient. Microbiology.

[37] Booth G.H., Robb J.A. (1968). Bacterial degradation of plasticised PVC-effect on some physical properties. J. Appl. Chem..

[38] Huang L., Zhu X., Zhou S., Cheng Z., Shi K., Zhang C., Shao H. (2021). Phthalic acid esters: Natural sources and biological activities. Toxins.

[39] Rijavec T., Strlič M., Cigić I.K. (2020). Plastics in heritage collections: Poly (vinyl chloride) degradation and characterization. Acta Chim. Slov..

[40] Ali S., Rehman A., Hussain S.Z., Bukhari D.A. (2023). Characterization of plastic degrading bacteria isolated from sewage wastewater. Saudi J. Biol. Sci..

[41] Magdy N., Maher M.S., Soliman A.M., Moussa T.A., Shehata H.M. (2026). A novel bacterial consortium isolated from long-term plastic-contaminated soil exhibits efficient biodegradation of polyvinyl chloride microplastics. Microb. Cell Fact..

[42] Xu Y., Xian Z.N., Yue W., Yin C.F., Zhou N.Y. (2023). Degradation of polyvinyl chloride by a bacterial consortium enriched from the gut of Tenebrio molitor larvae. Chemosphere.

[43] Rashmi M., Singh S. (2022). Microbial treatment of di (2-ethyl hexyl) phthalate by *Lysinibacillus xylanilyticus* Isolated from landfill Soil. Int. J. Plant Environ..

[44] Das G., Bordoloi N.K., Rai S.K., Mukherjee A.K., Karak N. (2012). Biodegradable and biocompatible epoxidized vegetable oil modified thermostable poly (vinyl chloride): Thermal and performance characteristics post biodegradation with *Pseudomonas aeruginosa* and *Achromobacter* sp.. J. Hazard. Mater..

[45] Giacomucci L., Raddadi N., Soccio M., Lotti N., Fava F. (2019). Polyvinyl chloride biodegradation by *Pseudomonas citronellolis* and *Bacillus flexus*. New Biotechnol..

[46] Wang D., Yu H., Liu X., Sun L., Liu X., Hu R., Wang C., Zhuge Y., Xie Z. (2024). The complete genome sequence of *Bacillus toyonensis* Cbmb3 with polyvinyl chloride-degrading properties. J. Xenobiot..

[47] Kucic Grgic D., Miloloza M., Lovrincic E., Kovacevic A., Cvetnic M., Ocelic Bulatovic V., Prevaric V., Bule K., Ukic S., Markic M. (2021). Bioremediation of MP-polluted waters using bacteria *Bacillus licheniformis*, *Lysinibacillus massiliensis*, and mixed culture of *Bacillus* sp. and *Delftia acidovorans*. Chem. Biochem. Eng..

[48] Pham V.H.T., Kim J., Chang S. (2024). A valuable source of promising extremophiles in microbial plastic degradation. Polymers.

[49] Zampolli J., Collina E., Lasagni M., Di Gennaro P. (2024). Insights into polyethylene biodegradative fingerprint of *Pseudomonas citronellolis* E5 and *Rhodococcus erythropolis* D4 by phenotypic and genome-based comparative analyses. Front. Bioeng. Biotechnol..

[50] Ghatge S., Yang Y., Ahn J.H., Hur H.G. (2020). Biodegradation of polyethylene: A brief review. Appl. Biol. Chem..

[51] Kumari P., Kumar A. (2023). Advanced oxidation process: A remediation technique for organic and non-biodegradable pollutant. Results Surf. Interfaces.

[52] Rachıd N.A., Güngör N.D. (2020). Microplastics in our planet: Source, distribution, effects and biodegradation. Eskişeh. Tek. Üniversitesi Bilim Teknol. Derg.-C Yaşam Bilim. Biyoteknoloji.

[53] Yadav M., Manderia S., Singh S., Deva M. (2022). Isolation and characterization of polyvinyl chloride (PVC) degrading bacteria from polluted sites of Gwalior city, MP, India. Nat. Environ. Pollut. Technol..

[54] Booth G.H., Cooper A.W., Robb J.A. (1968). Bacterial degradation of plasticized PVC. J. Appl. Bacteriol..

[55] Chen X., Stewart P.S. (2000). Biofilm removal caused by chemical treatments. Water Res..

[56] Osada F., Yoshioka T. (2012). Dechlorination of Polyvinyl Chloride in NaOH and NaOH/Ethylene Glycol Solution by Microwave Heating. The Development and Application of Microwave Heating.

[57] Rad M.M., Moghimi H., Azin E. (2022). Biodegradation of thermo-oxidative pretreated low-density polyethylene (LDPE) and polyvinyl chloride (PVC) microplastics by *Achromobacter denitrificans* Ebl13. Mar. Pollut. Bull..

[58] El-Kurdi N., El-Shatoury S., ElBaghdady K., Hammad S., Ghazy M. (2024). Biodegradation of polystyrene nanoplastics by *Achromobacter xylosoxidans* M9 offers a mealworm gut-derived solution for plastic pollution. Arch. Microbiol..

[59] Nyamjav I., Jang Y., Lee Y.E., Lee S. (2023). Biodegradation of polyvinyl chloride by *Citrobacter koseri* isolated from superworms (*Zophobas atratus* larvae). Front. Microbiol..

[60] Bayot M.L., Bragg B.N. (2024). Antimicrobial susceptibility testing. StatPearls.

[61] Jiang Y., Fu B., Wu W.M., Zhou C., Khan A., Zhang G., Salama E.S., Jeon B.H., Alreshidi M.A., Li C. (2025). Soil microbes in the Tibetan Plateau degrade polyvinyl chloride and harbor novel dehalogenase SerB. Environ. Int..

