# Supplementary material for: Characterization and GC–MS-Based Identification of Possible PVC Degradation Pathways of Bacteria for Sustainable Microplastic Bioremediation
Source: Microorganisms. 2026 Sep 21;14(9):2114. doi: 10.3390/microorganisms14092114 (PMC13609082; doi:10.3390/microorganisms14092114)
Supplement: Supplementary file 1 [file microorganisms-14-02114-s001.zip › microorganisms-4448911-supplementary.pdf]

Supplementary Table S1: Colony forming units (CFUs) enumeration of the current study bacteria at different dilution factors

Serial no.	Dilution factor	No. of colonies	CFUs/ml
1	Original	213	213
2	10^{-1}	40	4×10^{2}
3	10^{-2}	5	5×10^{2}

Supplementary Table S2: Morphological characterization of PVC-degrading isolates

Sr No.	Isolate Name	Shape	Size	Texture	Color	Edges
1	*Achromobacter xylosidans SBBPVC*1	Circular	Medium-large	Smooth	Pale yellow	Entire
2	*Bacillus licheniformis* SBBPVC2	Circular	Large	Dry	Off white	Undulate
3	*Pseudomonas citronellolis* SBBPVC3	Circular	Medium	Mucoid	Yellowish	Entire
4	*Pseudomonas stutzeri* SBBPVC4	Irregular	Medium	Dry	Cream to tan	Irregular
5	*Bacillus flexus* SBBPVC5	Circular	Large	Dry	White to pale yellow	Wavy

Supplementary Table S3: Absorbance values of bacterial isolates at 600 (nm) over 72 h' period

**Time (hrs)**	***A. xylosidans***		***B. licheniformis***		***P. citronellolis***		***P. stutzeri***		***B. flexus***	
	**Mean**	**SD**	**Mean**	**SD**	**Mean**	**SD**	**Mean**	**SD**	**Mean**	**SD**
**0**	0.237	0.006	0.22	0.0025	0.188	0.013	0.118	0.005	0.1	0.003
**3**	0.324	0.0065	0.362	0.0025	0.327	0.006	0.323	0.006	0.208	0.002
**6**	0.414	0.006	0.494	0.001	0.474	0.0025	0.352	0.0015	0.326	0.0025
**24**	0.534	0.0115	0.667	0.001	0.666	0.003	0.524	0.0145	0.442	0.0015
**27**	0.565	0.0015	0.69	0.0045	0.692	0.0015	0.542	0.005	0.452	0.009
**30**	0.568	0.016	0.694	0.0055	0.68	0.0075	0.544	0.0115	0.448	0.0145
**48**	0.523	0.003	0.667	0.008	0.657	0.013	0.511	0.016	0.433	0.002
**54**	0.433	0.003	0.592	0.0015	0.575	0.0015	0.444	0.0005	0.359	0.0245
**72**	0.35	0.003	0.513	0.009	0.514	0.0055	0.392	0.006	0.265	0.003

Supplementary Table S4: Growth phases of PVC degrading bacterial isolates measured based on OD^{600}

Strain	Lag phase (hrs.)	Log phase (hrs.)	Stationary phase (hrs.)	Death phase (hrs.)
A. xylosidans SBBPVC1	0-3	3< - 24	24< - 30	30< - onwards
B. licheniformis SBBPVC2	0-3	3< - 24	24< - 30	30< - onwards
P. citronellolis SBBPVC3	0-3	3< - 24	24< - 27	30< - onwards
P. stutzeri SBBPVC4	0-6	6< - 24	24< - 30	30< - onwards
B. flexus SBBPVC5	0-6	6< - 24	24< - 30	30< - onwards

Supplementary Table S5: Statistical analysis of growth curve study via time-point-by-time-point comparison and growth parameter extraction

Isolate	Time (h)	Mean_OD600	SD	Control_OD600	t-statistic	p-value	Significance
A. xylosidans SBBPVC1	0	0.237	0.006	0.108	37.24	0.0004	***
A. xylosidans SBBPVC1	3	0.324	0.0065	0.102	59.16	0.0001	***
A. xylosidans SBBPVC1	6	0.414	0.006	0.112	87.18	<0.0001	***
A. xylosidans SBBPVC1	24	0.534	0.0115	0.1041	64.75	0.0001	***
A. xylosidans SBBPVC1	27	0.565	0.0015	0.093	545.02	<0.0001	***
A. xylosidans SBBPVC1	30	0.568	0.016	0.114	49.15	0.0002	***
A. xylosidans SBBPVC1	48	0.523	0.003	0.102	243.06	<0.0001	***
A. xylosidans SBBPVC1	54	0.433	0.003	0.114	184.17	<0.0001	***
A. xylosidans SBBPVC1	72	0.35	0.003	0.086	152.42	<0.0001	***
B. licheniformis SBBPVC2	0	0.22	0.0025	0.108	77.6	<0.0001	***
B. licheniformis SBBPVC2	3	0.362	0.0025	0.102	180.13	<0.0001	***
B. licheniformis SBBPVC2	6	0.494	0.001	0.112	661.64	<0.0001	***
B. licheniformis SBBPVC2	24	0.667	0.001	0.1041	974.97	<0.0001	***
B. licheniformis SBBPVC2	27	0.69	0.0045	0.093	229.79	<0.0001	***
B. licheniformis SBBPVC2	30	0.694	0.0055	0.114	182.65	<0.0001	***
B. licheniformis SBBPVC2	48	0.667	0.008	0.102	122.33	<0.0001	***
B. licheniformis SBBPVC2	54	0.592	0.0015	0.114	551.95	<0.0001	***
B. licheniformis SBBPVC2	72	0.513	0.009	0.086	82.18	<0.0001	***
P. citronellolis SBBPVC3	0	0.188	0.013	0.108	10.66	0.0043	**
P. citronellolis SBBPVC3	3	0.327	0.006	0.102	64.95	0.0001	***
P. citronellolis SBBPVC3	6	0.474	0.0025	0.112	250.8	<0.0001	***
P. citronellolis SBBPVC3	24	0.666	0.003	0.1041	324.41	<0.0001	***
P. citronellolis SBBPVC3	27	0.692	0.0015	0.093	691.67	<0.0001	***
P. citronellolis SBBPVC3	30	0.68	0.0075	0.114	130.71	<0.0001	***
P. citronellolis SBBPVC3	48	0.657	0.013	0.102	73.95	<0.0001	***

P. citronellolis SBBPVC3	54	0.575	0.0015	0.114	532.32	<0.0001	***
P. citronellolis SBBPVC3	72	0.514	0.0055	0.086	134.79	<0.0001	***
P. stutzeri SBBPVC4	0	0.118	0.005	0.108	3.46	0.0371	*
P. stutzeri SBBPVC4	3	0.323	0.006	0.102	63.8	0.0001	***
P. stutzeri SBBPVC4	6	0.352	0.0015	0.112	277.13	<0.0001	***
P. stutzeri SBBPVC4	24	0.524	0.0145	0.1041	50.16	0.0002	***
P. stutzeri SBBPVC4	27	0.542	0.005	0.093	155.54	<0.0001	***
P. stutzeri SBBPVC4	30	0.544	0.0115	0.114	64.76	0.0001	***
P. stutzeri SBBPVC4	48	0.511	0.016	0.102	44.28	0.0003	***
P. stutzeri SBBPVC4	54	0.444	0.0005	0.114	1143.15	<0.0001	***
P. stutzeri SBBPVC4	72	0.392	0.006	0.086	88.33	<0.0001	***
B. flexus SBBPVC5	0	0.1	0.003	0.108	-4.62	0.9781	ns
B. flexus SBBPVC5	3	0.208	0.002	0.102	91.8	<0.0001	***
B. flexus SBBPVC5	6	0.326	0.0025	0.112	148.26	<0.0001	***
B. flexus SBBPVC5	24	0.442	0.0015	0.1041	390.17	<0.0001	***
B. flexus SBBPVC5	27	0.452	0.009	0.093	69.09	0.0001	***
B. flexus SBBPVC5	30	0.448	0.0145	0.114	39.9	0.0003	***
B. flexus SBBPVC5	48	0.433	0.002	0.102	286.65	<0.0001	***
B. flexus SBBPVC5	54	0.359	0.0245	0.114	17.32	0.0017	**
B. flexus SBBPVC5	72	0.265	0.003	0.086	103.35	<0.0001	***

Supplementary Table S6: Statistical analysis of antibiotic sensitivity profiling of PVC degrading isolates via One-Sample T-test

Isolate	Dose (µL)	Mean ± SD (mm)	t-statistic	p-value	Significance
A. xylosoxidans SBBPVC1	5	9.50 ± 0.71	19.00	0.0167	*
A. xylosoxidans SBBPVC1	10	11.50 ± 0.71	23.00	0.0138	*
A. xylosoxidans SBBPVC1	15	14.50 ± 0.71	29.00	0.0110	*
B. licheniformis SBBPVC2	5	10.50 ± 0.71	21.00	0.0151	*
B. licheniformis SBBPVC2	10	12.50 ± 0.71	25.00	0.0127	*
B. licheniformis SBBPVC2	15	15.50 ± 0.71	31.00	0.0103	*
P. citronellolis SBBPVC3	5	9.50 ± 0.71	19.00	0.0167	*
P. citronellolis SBBPVC3	10	10.50 ± 0.71	21.00	0.0151	*
P. citronellolis SBBPVC3	15	21.00 ± 1.41	21.00	0.0151	*
P. stutzeri SBBPVC4	5	1.50 ± 0.71	3.00	0.1024	ns
P. stutzeri SBBPVC4	10	6.50 ± 0.71	13.00	0.0244	*
P. stutzeri SBBPVC4	15	8.50 ± 0.71	17.00	0.0187	*
B. flexus SBBPVC5	5	2.50 ± 0.71	5.00	0.0628	ns
B. flexus SBBPVC5	10	7.00 ± 1.41	7.00	0.0452	*
B. flexus SBBPVC5	15	10.50 ± 0.71	21.00	0.0151	*

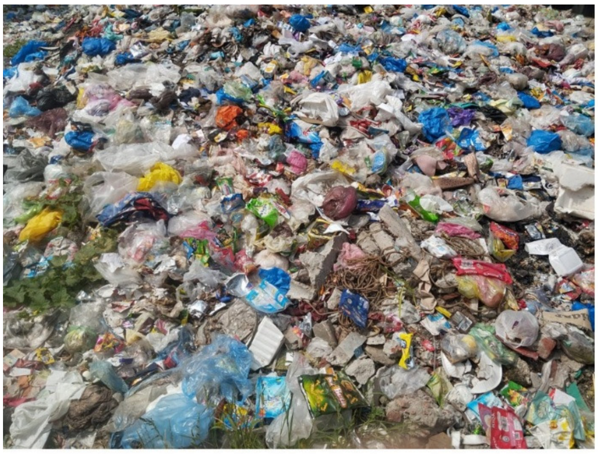

(A)

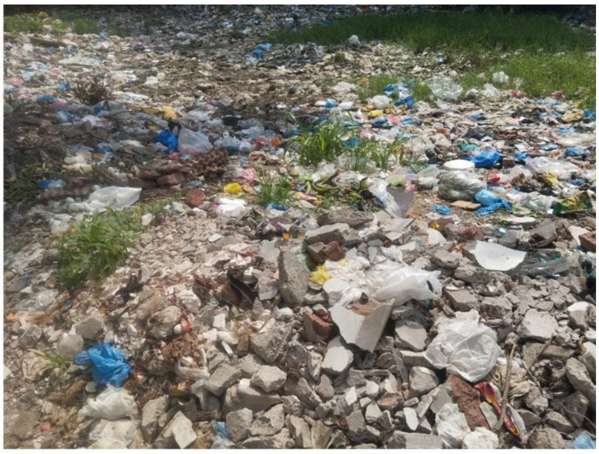

(B)

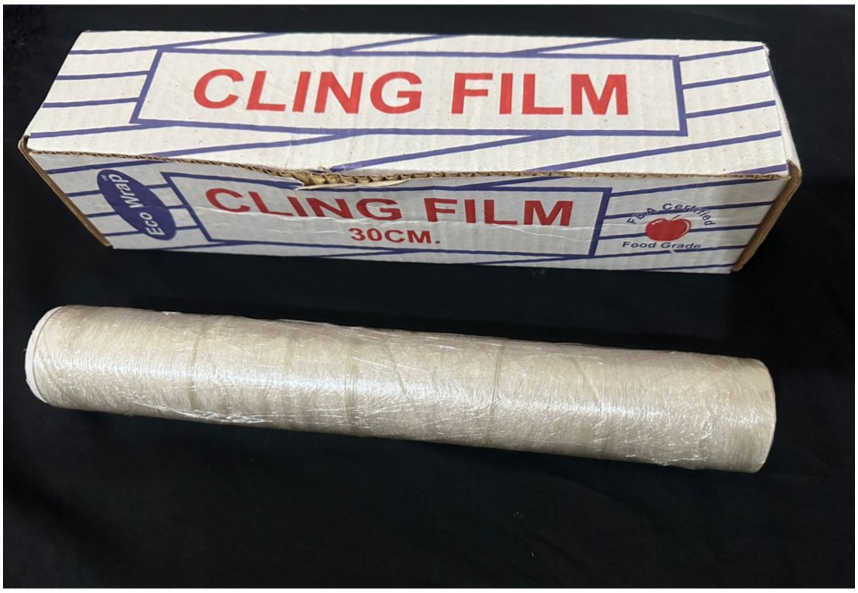

(C)

Supplementary Figure S1: Sites in Multan selected for soil sample collection for isolation of PVC degrading bacteria (A and B) and Cling foil used as PVC substrate for current study documented bacteria (C)

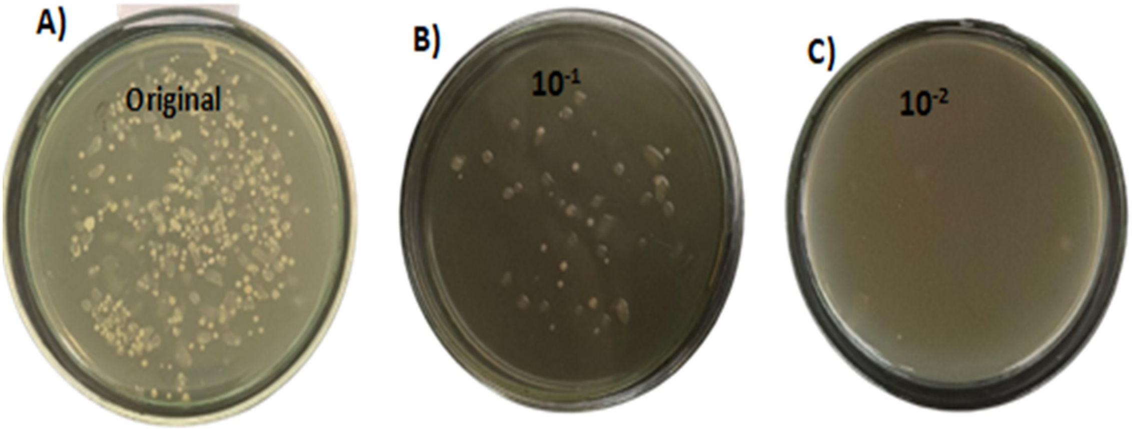

Supplementary Figure S2: CFUs of PVC-degrading bacteria isolated by the serial dilution method

a) Colonies formed in the original plate b) Colonies formed in10^{-1} dilution c) Colonies obtained in 10^{-2} dilution

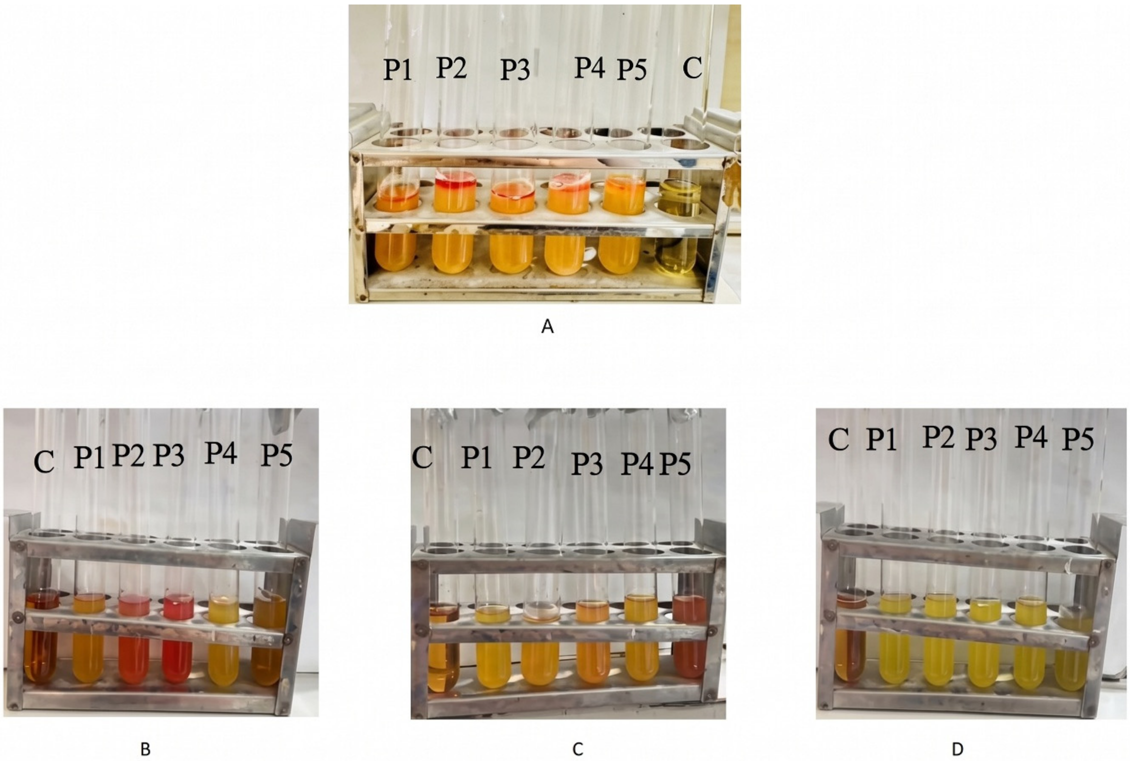

Supplementary Figure S3: Biochemical characterization of current study bacteria via indole acetic acid (IAA) production and glucose (Glu), sucrose (SUC) and lactose (LAC) fermentation tests

A: IAA test, B: GLU test, C: SUC test, D: LAC test, P1: *A. xylosoxidans* SBBPVC1, P2: *B. licheniformis* SBBPVC2, P3: *P. citronellolis* SBBPVC3, P4: *P. stutzeri* SBBPVC4, P5: *B. flexus* SBBPVC5

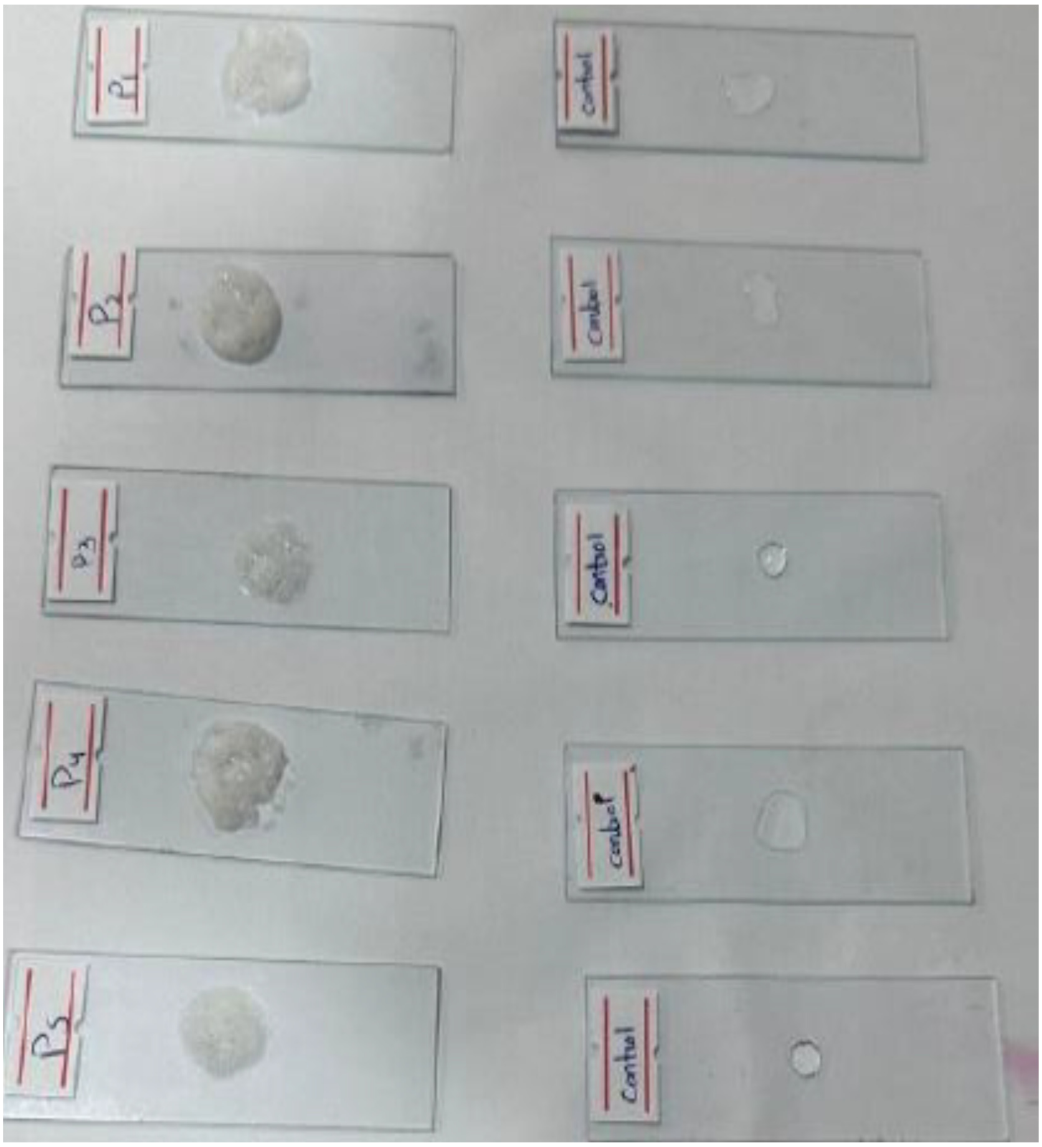

(A)

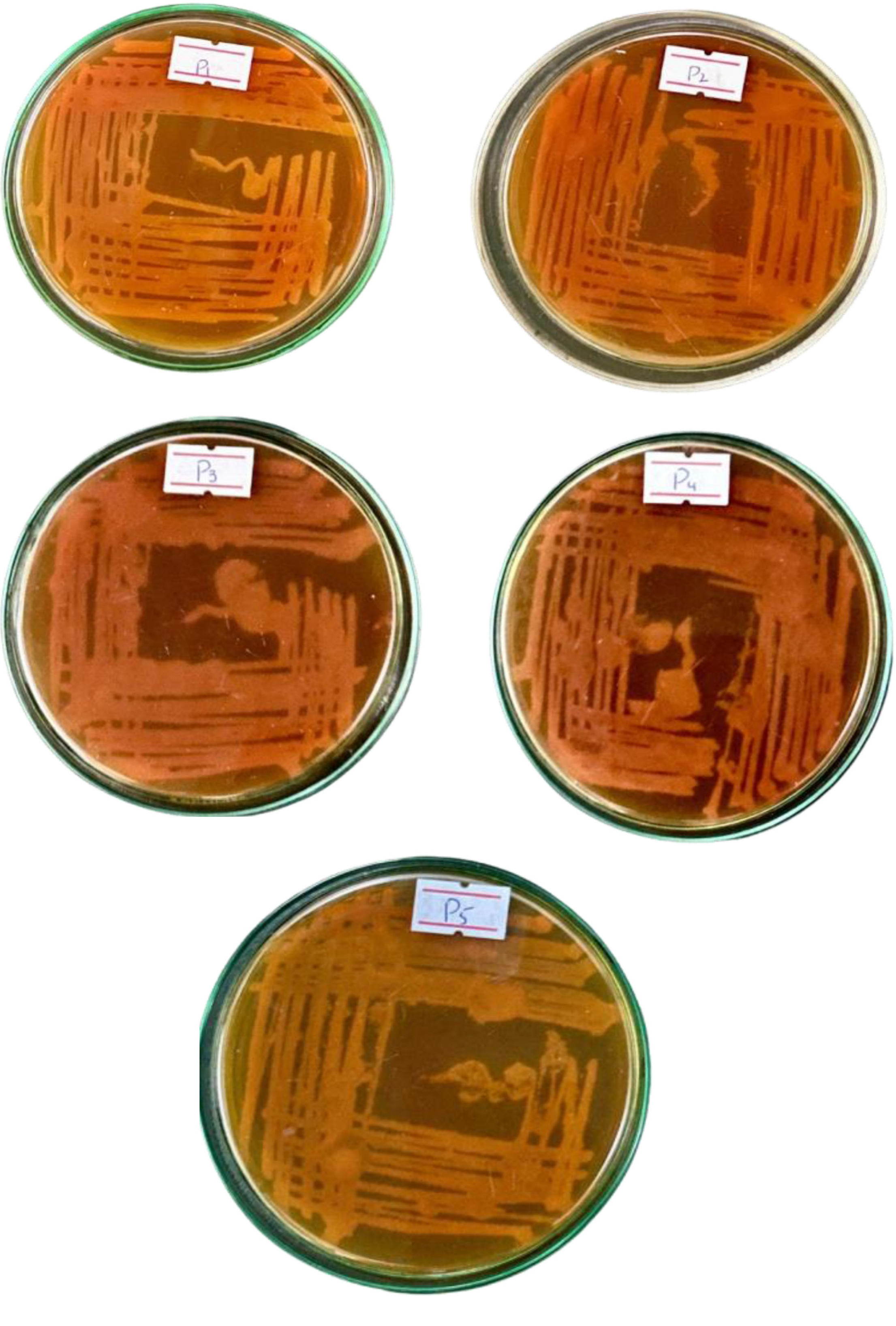

(B)

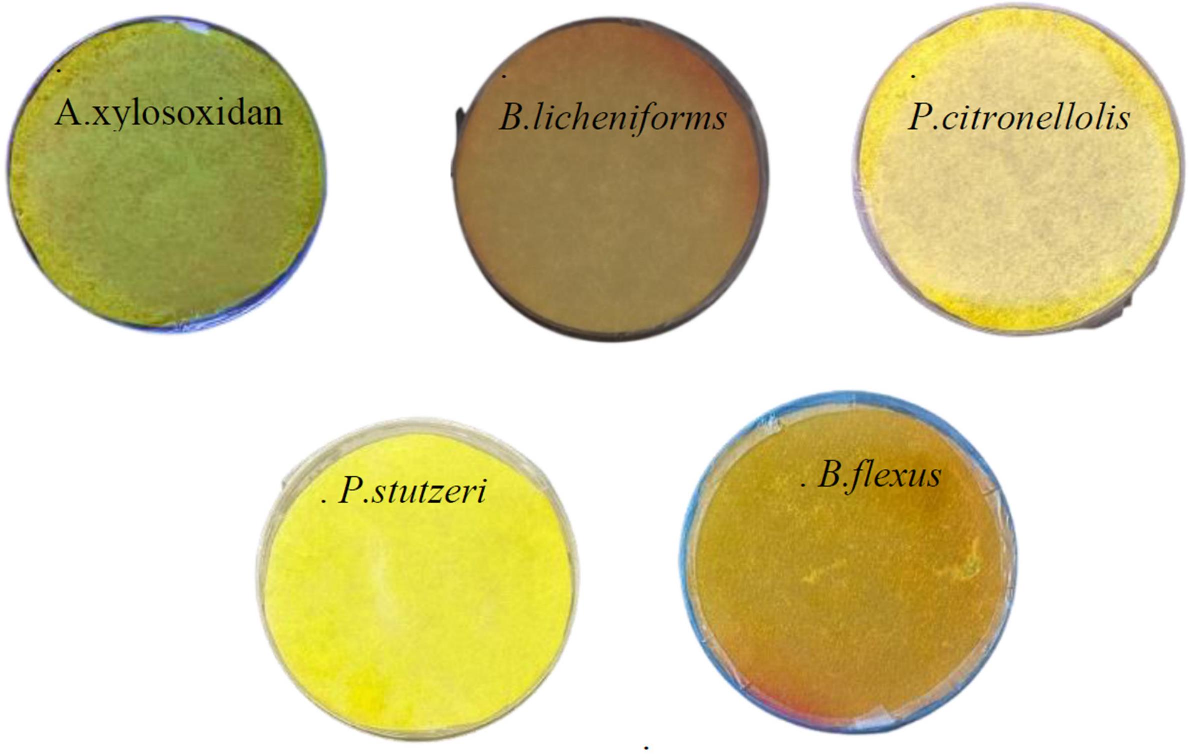

(C)

Supplementary Figure S4: Biochemical characterization of current study bacteria via catalase (CAT), mannitol sorbitol agar (MSA) and hydrogen cyanide (HCN) production tests

A: CAT test, B: MSA test, C: HCN test, P1: *A. xylosoxidans* SBBPVC1, P2: *B. licheniformis* SBBPVC2, P3: *P. citronellolis* SBBPVC3, P4: *P. stutzeri* SBBPVC4, P5: *B. flexus* SBBPVC5

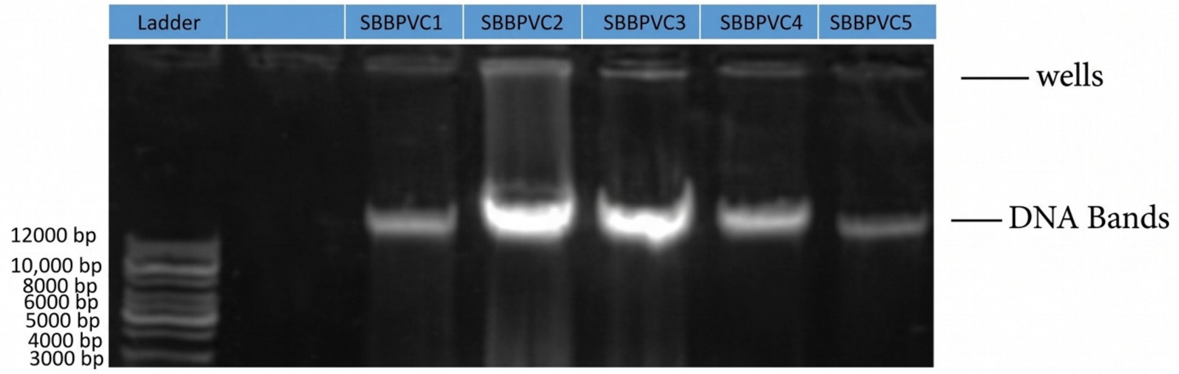

(A)

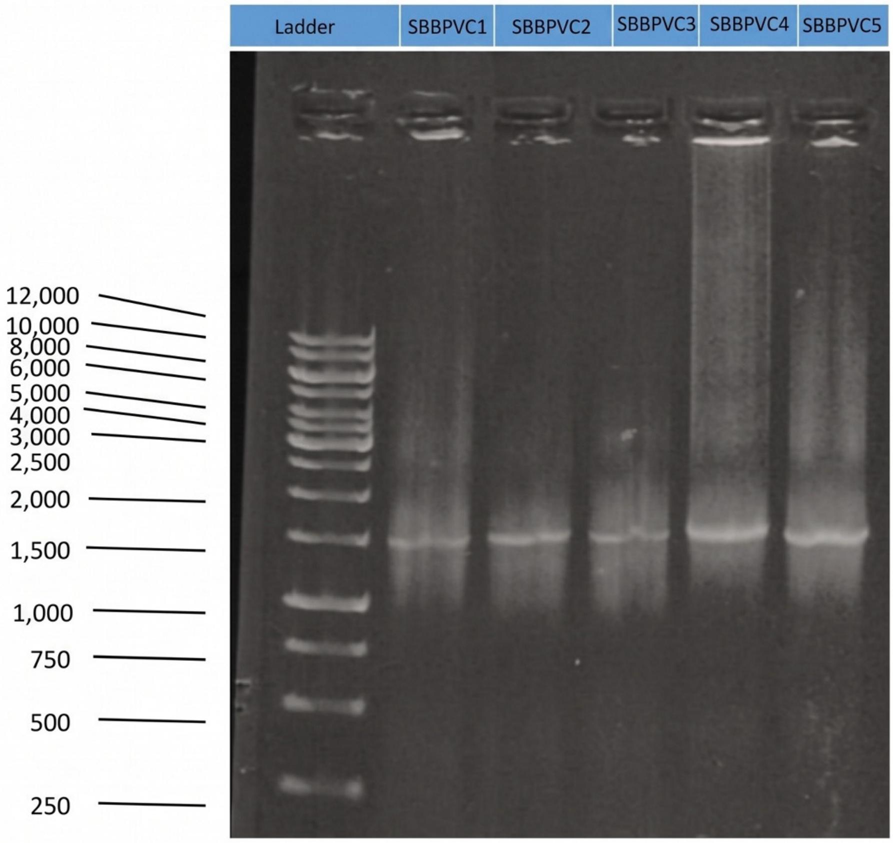

(B)

Supplementary Figure S5: Agarose gel electrophoresis showing (A) extracted genomic DNA and (B) PCR amplicons of current study PVC degrading bacteria
